# Bibliometric development of *Naunyn–Schmiedeberg’s Archives of Pharmacology*

**DOI:** 10.1007/s00210-022-02307-2

**Published:** 2022-10-25

**Authors:** Leah B. Dats, Florentin von Haugwitz, Roland Seifert

**Affiliations:** 1 Institute of Pharmacology, Hannover Medical School, Carl-Neuberg-Str. 1, 30625 Hannover, Germany; 2 Nova School of Business and Economics Carcavelos Campus, Rua da Holanda, no. 1, 2775-405 Carcavelos, Portugal

**Keywords:** Meta-data analysis, Bibliometric development, COVID-19 pandemic, Historical development, Research topics ⋅ *zeitgeist*

## Abstract

Motivated by the 150-year anniversary of the *Naunyn–Schmiedeberg’s Archives of Pharmacology* in 2023, we studied the bibliometric development of the journal. We evaluated data from Editorial Reports, Clarivate, and Springer Nature databases. Several parameters representing the journal’s performance, such as the impact factor and social impact, were analyzed over the years. We analyzed the journal’s meta-data and wrote an algorithm to retrieve cities and countries of origin. We could see a decrease in publications from Germany and an increase in papers from Brazil, China, Egypt, and Iran during the last years. The decrease in publications from Germany is probably a *zeitgeist* effect because this country places a strong emphasis on high-impact factor papers for academic promotion and winning grants. Germany was the country with the most publications throughout the 100 most-cited articles. Most of these articles were published between 1970 and 1990, when neurotransmitters were the most published topic. Klaus Starke (Freiburg) and Manfred Göthert (Bonn) were prominent drivers of this field. The most common topics nowadays are “Drugs for the Treatment of Malignant Tumor Diseases” and “Immunopharmacology.” The internationality of the journal substantially increased after introduction of English as mandatory language in the 1970s. The journal also experienced substantial COVID-19 pandemic-related effects. This paper is not only of relevance for the field of pharmacology but for science in general in the sense that *Naunyn–Schmiedeberg’s Archives of Pharmacology* is a case study for profound changes in a traditional scientific journal, requiring permanent adjustment by editors, referees, publisher, authors, and readers alike. The development of the journal has been strongly influenced by historic and political developments, cultural attitudes (*zeitgeist*), language changes, global changes in research topics, and eminent individuals who published many papers in *Naunyn–Schmiedeberg’s Archives of Pharmacology*.

**Supplementary Information:**

The online version contains supplementary material available at 10.1007/s00210-022-02307-2.

## Introduction

*Naunyn–Schmiedeberg’s Archives of Pharmacology* is the official journal of the German Society of Experimental and Clinical Pharmacology and Toxicology (DGPT), founded in 1873 (https://www.springer.com/journal/210/?IFA; last accessed August 2nd, 2022). It is the oldest journal in this field of science.

Founded by Bernhard Naunyn, Edwin Klebs, and Oswald Schmiedeberg, the first volume was printed by the “Vogel” publishing company from Leipzig on February 14th, 1873. Naunyn and Schmiedeberg met in Dorpat earlier, where both had professorships (Starke 1998). They later engaged Klebs to join their cause in creating a scientific magazine, which combined experimental studies from practical medicine with knowledge from theoretical medicine. Historically the journal was called “Archiv für Experimentelle Pathologie und Pharmakologie,” which changed several times during the decades and was later renamed to the current known title (Starke 1998).

On the 125th anniversary of *Naunyn–Schmiedeberg’s Archives of Pharmacology* in 1998, Starke (1998) published an extensive history of the journal. The focus of the review was the history of the journal and analysis of the scientific contributions of individual scientists. The 150th anniversary of *Naunyn–Schmiedeberg’s Archives of Pharmacology* in 2023 motivated us to investigate the journal more from a numerical perspective, focusing on bibliometric parameters. Our data yield a multi-facetted picture showing that editing a scientific journal is a cultural human activity that is influenced by many more factors more than just scientific data.

## Materials and methods

### Editorial analysis

To investigate developments of the journal between 2006 and 2021, we analyzed the Editorial Reports of *Naunyn–Schmiedeberg’s Archives of Pharmacology*. We included all Editorial Reports available. The Clarivate database (https://clarivate.com, last accessed May 30th, 2022) was used to complete our data records. The Clarivate database was also used to identify the authors with the most publications in the journal. We named the top-15 authors and assigned general research fields for each author by researching them on Pubmed (https://pubmed.ncbi.nlm.nih.gov, last accessed June 9th, 2022). We used Excel to create charts and tables.

### Most-cited articles

Using the Clarivate database (https://clarivate.com, last accessed May 30th, 2022), we identified the journal’s most-cited articles. The most-cited articles represent the journal’s highest cited publications. The ranking is solely based on number of citations recorded until the data retrieval date, December 8th, 2021. Only documents published after 1947 are included, due to missing information for earlier publications in the database. We decided to focus on the 100 most-cited articles, analyzing the topics, article types, citations, and cities and countries of origin. The article titles were used to assign research topics, based on the chapters of the textbook “Basic Knowledge of Pharmacology” (Seifert 2019). Each article was assigned to one chapter. We introduced “Purinergic System” and “Substance P” as additional topics, which are not chapters of the textbook. Especially the most-cited article was investigated more closely, including the analysis of authors, research organization, number of citations, and number of registered accesses since 2013. We extracted data from Clarivate using Excel charts.

### Zero-cited articles

We analyzed articles with zero citations published from 2015 to 2022. First, we defined the document types. We focused on original articles and review articles, analyzing topics as well as researching the author’s region of origin. We repeated the same approach as introduced before and used “Basic Knowledge of Pharmacology” (Seifert 2019) and its chapters to assign topics of an article. For the geographical analysis, we listed the cities and countries of origin. We used SpringerLink to research origins and used the first affiliation listed (https://www.springer.com/journal/210, last accessed May 30th, 2022). We received the data listed in Excel sheets. Information such as article titles, authors, and citations was provided.

### Metadata

The metadata of *Naunyn–Schmiedeberg’s Archives of Pharmacology* were extracted from the journal website (https://www.springer.com/journal/210, last accessed May 30th, 2022).

### Metadata-publication languages

Publication languages were studied from 1947 to 1976. Although English was appointed the official publication language for the journal in 1972, the last articles in another language were published in 1975. Publication languages in these years were both German and English. We listed the annual numbers and percentages of papers in tables and designed charts using Excel.

### Metadata topics

We analyzed the topics of articles published in the years 1970, 1980, 1990, 2000, 2010, and 2020. We used the chapters from the textbook “Basic Knowledge of Pharmacology” (Seifert 2019) and defined the theme by title of articles. Each article was assigned to one chapter. We introduced “Purinergic System,” “Substance P,” “Editorial Analysis,” and “Drugs for Treatment of Parasitic Infections” as additional topics, which are not chapters of the textbook. Annual shares were calculated, and numbers were rounded up to 0.5% in this calculation. We worked with Excel to create charts.

### Metadata country of research organization

The geographical analysis of publications was the most challenging part of the meta-data analysis, due to wrongly appointed or missing data in the database. This false information had to be detected, selected, and substituted. We chose to limit our study to analyzing 30 years, 1990–2020. During this period, we could properly appoint geographical origins and see much less mistakes in the database, which we had to rule out. We analyzed origins by “City of Research Organization” and “Country of Research Organization,” using the corresponding author as our reference. A city was assigned to the country it was part of when the article was published. Modern city names were used. The Python package was used to check if the city or country name was in English language. If not, German city and country names were translated into the English language, after separation from the associated author and the research organization (https://www.deepl.com/de/blog/announcing-python-client-library-for-deepl-api, last accessed June 27th, 2022). Geopy is a Python package, which contains a database listing all cities and countries in English language. This database was used to unify the names of cities and countries. In addition, the algorithm can detect spelling mistakes in the names and correct these automatically (https://geopy.readthedocs.io/en/latest/, last accessed June 27th, 2022). If city or country names were missing or showed significant deviation, the correct translation of a name was researched manually (https://www.springer.com/journal/210, last accessed May 30th, 2022). Analyses focusing on continents, countries, and cities of origin were made. Trends were visualized over time as well as rising publication origins. Publication hotspots in Germany were identified and listed.

## Results and discussion

### Editorial analysis

Compared to 2019, the number of submissions increased by 36% in 2020. In 2021, the number of submissions then decreased by 23%. The substantial increase in articles submitted in 2020 could be an effect of the COVID-19 pandemic, with fewer new projects being initiated in laboratories and more time to publish earlier collected data (Gao et al. 2021). In 2021, the stock of still unpublished data was used up, resulting in decreased submission numbers. From 2007 to 2021, the number of downloads increased by 3.5-fold. In March 2020, marking the first COVID-19-pandemic lockdown in many countries, downloads increased by almost 40%, when compared with February 2020. While comparing monthly downloads over the years, we found May to be the most active month (spring high). May is followed by October and November (fall high). August and September are the months with the lowest number of downloads (summer low), which leads us to conclude that in contrast to general assumption, scientists are not working year-round with the same intensity but take time off during summer.

**Fig. 1:**
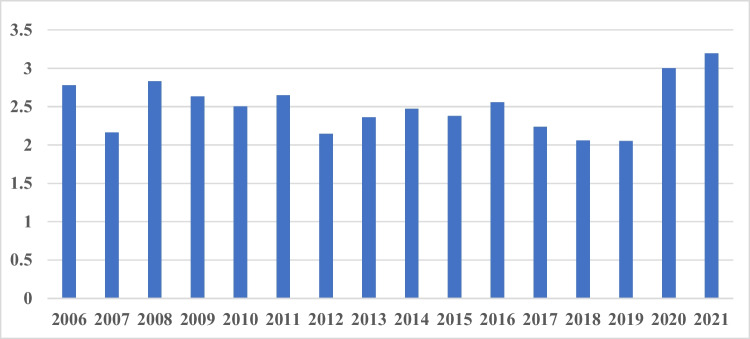
Journal impact factor

The journal impact factor is a factor, which is calculated and published by the company Clarivate (https://clarivate.com/webofsciencegroup/essays/impact-factor/, last accessed June 7th, 2022). It was developed to represent frequently cited papers and compare journals by their citations. Although the impact factor has been broadly criticized an unsuitable bibliometric tool to asses research quality and scientific contributions of individual scientists (see, e.g., https://pubmed.ncbi.nlm.nih.gov/28447650/, last accessed August 4th, 2022), nonetheless, it is broadly used as parameter in the scientific world, determining a journal’s “success” and standing. Authors still try to publish in journals with the highest impact factor possible, because they seemingly have a better reputation and a higher standing, potentially having better chances in job offers (Couch 2020). The impact factor of *Naunyn-Schmiedebergs’ Archives of Pharmacology* showed some ups and downs over the years and reached a peak of 3.195 in 2021 (Fig. 1).

Journals publishing exclusively reviews or many reviews tend to have a higher impact factor than journals focusing on original articles (see Clarivate database). *Naunyn–Schmiedeberg’s Archives of Pharmacology* clearly belongs into the second category (see discussion below). A recent analysis showed that the number of authors from Germany dropped substantially over the past two decades (Zehetbauer et al. 2022). The major reason for this drop is probably a cultural change in Germany taking place in the late 1990s, placing strong emphasis on a high impact factor for securing tenured academic positions and grant support (Zehetbauer et al. 2022). In line with this trend, not only the number of authors from Germany dropped over the past decades but also the number of submissions from Germany (see discussion below). Thus, one may be tempted to conclude that for many pharmacologists in Germany, the impact factor of *Naunyn–Schmiedeberg’s Archives of Pharmacology* is “too low” to stimulate high-quality submissions. In contrast to this preconceived opinion, papers published by German pharmacologists in *Naunyn–Schmiedeberg’s Archives of Pharmacology* may receive high international visibility as evidenced by the high number of citations of the top-100 papers of this journal (see discussion below and Table 1).

**Table 1:** 100 most-cited articles in Naunyn–Schmiedeberg’s Archives of Pharmacology

Rank	Times cited, all databases	Article title	Authors	Volume (Year)	Start page	End page
1	1157	Substance-P as neurogenic mediator of antidromic vasodilation and neurogenic plasma extravasation	Lembeck, F; Holzer, P	310 (1979)	175	183
2	747	Extracellular metabolism of ATP and other nucleotides	Zimmermann, H	362 (2000)	299	309
3	716	Effects of alpha-adrenoceptor agonists and antagonists in a maze-exploration model of fear-motivated behavior	Handley, Sl; Mithani, S	327 (1984)	1	5
4	668	Identity of inhibitory presynaptic 5-hydroxytryptamine (5-HT) autoreceptors in the rat-brain cortex with 5-HT1B binding-sites	Engel, G; Göthert, M; Hoyer, D; Schlicker, E; Hillenbrand, K	332 (1986)	1	7
5	666	Simultaneous measurement of tyrosine and tryptophan hydroxylase-activities in brain in-vivo using an inhibitor of aromatic amino-acid decarboxylase	Carlsson, A; Atack, CV; Lindqvist, M; Kehr, W; Davis, JN	275 (1972)	153	+
6	613	Cytochrome P450 2D6: overview and update on pharmacology, genetics, biochemistry	Zanger, UM; Raimundo, S; Eichelbaum, M	369 (2004)	23	37
7	584	Relative presynaptic and postsynaptic potencies of alpha-adrenoceptor agonists in rabbit pulmonary-artery	Starke, K; Endo, T; Taube, HD	291 (1975)	55	78
8	529	The binding spectrum of narcotic analgesic drugs with different agonist and antagonist properties	Magnan, J; Paterson, SJ; Tavani, A; Kosterlitz, HW	319 (1982)	197	205
9	481	Zur Frage der zentralen Übertragung afferenter Impulse.3. Das Vorkommen und die Bedeutung der Substanz-P in den dorsalen Wurzeln des Rückenmarks	Lembeck, F	219 (1953)	197	213
10	469	Dopamine autoreceptors—pharmacological characterization by microiontophoretic single cell recording studies	Aghajanian, GK; Bunney, BS	297 (1977)	1	7
11	460	Constitutive activity of G-protein-coupled receptors: cause of disease and common property of wild-type receptors	Seifert, R; Wenzel-Seifert, K	366 (2002)	381	416
12	449	Vascular protein leakage in various tissues induced by substance-P, capsaicin, bradykinin, serotonin, histamine and by antigen challenge	Saria, A; Lundberg, JM; Skofitsch, G; Lembeck, F	324 (1983)	212	218
13	439	Structure and function of adenosine receptors and their genes	Fredholm, BB; Arslan, G; Halldner, L; Kull, B; Schulte, G; Wasserman, W	362 (2000)	364	374
14	435	(+ /)(Iodo-125)cyanopindolol, a new ligand for beta-adrenoceptors—identification and quantitation of subclasses of beta-adrenoceptors in guinea-pig	Engel, G; Hoyer, D; Berthold, R; Wagner, H	317 (1981)	277	285
15	433	Possible common mechanism of action of antidepressant treatments—reduction in sensitivity of noradrenergic cyclic-AMP generating system in rat limbic forebrain	Vetulani, J; Stawarz, RJ; Dingell, JV; Sulser, F	293 (1976)	109	114
16	431	Different alpha-adrenoreceptors in central nervous-system mediating biochemical and functional effects of clonidine and receptor blocking-agents	Anden, NE; Grabowska, M; Strombom, U	292 (1976)	43	52
17	429	Comparative pharmacology of human adenosine receptor subtypes—characterization of stably transfected receptors in CHO cells	Klotz, KN; Hessling, J; Hegler, J; Owman, C; Kull, B; Fredholm, BB; Lohse, MJ	357 (1998)	1	9
18	418	MDL-72222—a potent and highly selective antagonist at neuronal 5-hydroxytryptamine receptors	Fozard, JR	326 (1984)	36	44
19	410	8-Cyclopentyl-1,3-dipropylxanthine (DPCPX)—a selective high-affinity antagonist radioligand for a1 adenosine receptors	Lohse, MJ; Klotz, KN; Lindenbornfotinos, J; Reddington, M; Schwabe, U; Olsson, RA	336 (1987)	204	210
20	405	Molecular pharmacology of P2Y-receptors	Von Kügelgen, I; Wetter, A	362 (2000)	310	323
21	396	Binding of the A1-selective adenosine antagonist 8-cyclopentyl-1,3-dipropylxanthine to rat-brain membranes	Bruns, RF; Fergus, JH; Badger, EW; Bristol, JA; Santay, LA; Hartman, JD; Hays, SJ; Huang, CC	335 (1987)	59	63
22	384	Evidence for cyclic GMP-mediated relaxant effects of nitro-compounds in coronary smooth-muscle	Kukovetz, WR; Holzmann, S; Wurm, A; Poch, G	310 (1979)	129	138
23	384	Nitric-oxide synthase—expression and expressional control of the 3 isoforms	Förstermann, U; Kleinert, H	352 (1995)	351	364
24	360	Dopaminergic-neurons—invivo system for measuring drug interactions with presynaptic receptors	Walters, JR; Roth, RH	296 (1976)	5	14
25	355	The possible existence of multiple receptors for substance-P	Lee, CM; Iversen, Ll; Hanley, MR; Sandberg, BEB	318 (1982)	281	287
26	355	Comparison of effects of clonidine on presynaptic and postsynaptic adrenoceptors in rabbit pulmonary-artery—alpha-sympathomimetic inhibition of neurogenic vasoconstriction	Starke, K; Montel, H; Gayk, W; Merker, R	285 (1974)	133	150
27	351	NG-nitro-l-arginine (N-5-[imino(nitroamino)methyl]-l-ornithine) impairs endothelium-dependent dilations by inhibiting cytosolic nitric-oxide synthesis from l-arginine	Mülsch, A; Busse, R	341 (1990)	143	147
28	350	Presynaptic receptor systems on noradrenergic neurons of rat-brain	Taube, HD; Starke, K; Borowski, E	299 (1977)	123	141
29	349	Presynaptic and postsynaptic effects of yohimbine stereoisomers on noradrenergic transmission in the pulmonary-artery of the rabbit	Weitzell, R; Tanaka, T; Starke, K	308 (1979)	127	136
30	349	Catecholamine receptor agonists—effects on motor activity and rate of tyrosine hydroxylation in mouse-brain	Strombom, U	292 (1976)	167	176
31	334	Differential-effects of capsaicin on the content of somatostatin, substance-P, and neurotensin in the nervous-system of the rat	Gamse, R; Leeman, SE; Holzer, P; Lembeck, F	317 (1981)	140	148
32	327	A 3-state model of the benzodiazepine receptor explains the interactions between the benzodiazepine antagonist RO 15–1788, benzodiazepine tranquilizers, beta-carbolines, and phenobarbitone	Polc, P; Bonetti, EP; Schaffner, R; Haefely, W	321 (1982)	260	264
33	327	Pharmacogenetics of paraoxonases: a brief review	Draganov, DI; La Du, BN	369 (2004)	78	88
34	325	Effects of a selective 5-HT-reuptake blocker, citalopram, on the sensitivity of 5-HT autoreceptors—electrophysiological studies in the rat-brain	Chaput, Y; Demontigny, C; Blier, P	333 (1986)	342	348
35	312	Differentiation of cardiac chronotropic and inotropic effects of beta-adrenoceptor agonists	Carlsson, E; Dahlof, CG; Hedberg, A; Persson, H; Tangstrand, B	300 (1977)	101	105
36	312	Effects of adenosine on adrenergic neurotransmission—prejunctional inhibition and postjunctional enhancement	Hedqvist, P; Fredholm, BB	293 (1976)	217	223
37	308	Effect of dopamine receptor agonists and antagonists on release of dopamine in rabbit caudate-nucleus invitro	Starke, K; Reimann, W; Zumstein, A; Hertting, G	305 (1978)	27	36
38	308	Alpha sympathomimetic inhibition of adrenergic and cholinergic transmission in rabbit heart	Starke, K	274 (1972)	18	&
39	308	The 5-HT1A receptor agonist, 8-OH-DPAT, preferentially activates cell body 5-HT autoreceptors in rat-brain invivo	Hjorth, S; Magnusson, T	338 (1988)	463	471
40	307	Dependence of 5-HT and catecholamine synthesis on concentrations of precursor amino-acids in rat-brain	Carlsson, A; Lindqvist, M	303 (1978)	157	164
41	300	Characterization of adenosine receptors in rat-brain by (-)[H-3]n-6-phenylisopropyladenosine	Schwabe, U; Trost, T	313 (1980)	179	187
42	295	Method for determination of 3,4-dihydroxyphenylalanine (DOPA) in brain	Kehr, W; Carlsson, A; Lindqvist, M	274 (1972)	273	+
43	294	The gastrointestinal prokinetic benzamide derivatives are agonists at the non-classical 5-HT receptor (5-HT4) positively coupled to adenylate-cyclase in neurons	Dumuis, A; Sebben, M; Bockaert, J	340 (1989)	403	410
44	290	The use of nitric oxide donors in pharmacological studies	Feelisch, M	358 (1998)	113	122
45	283	Peristaltic reflex—analysis of nerve pathways and their pharmacology	Costa, M; Furness, JB	294 (1976)	47	60
46	282	A comparison of pre-junctional and post-junctional potencies of several alpha-adrenoceptor agonists in the cardiovascular-system and anococcygeus muscle of the rat—evidence for 2 types of post-junctional alpha-adrenoceptor	Docherty, JR; Mcgrath, JC	312 (1980)	107	116
47	271	Pharmacogenomics of human OATP transporters	König, J; Seithel, A; Gradhand, U; Fromm, MF	372 (2006)	432	443
48	270	Melatonin Receptor Antagonists That Differentiate Between The Human Mel(1a), And Mel(1b) Recombinant Subtypes Are Used To Assess The Pharmacological Profile Of The Rabbit Retina ML(1) Presynaptic Heteroreceptor	Dubocovich, Ml; Masana, MI; Iacob, S; Sauri, DM	355 (1997)	365	375
49	270	Histamine H-3 receptor-mediated inhibition of serotonin release in the rat-brain cortex	Schlicker, E; Betz, R; Göthert, M	337 (1988)	588	590
50	267	Adenosine receptors and their ligands	Klotz, KN	362 (2000)	382	391
51	259	Inotropic and electrophysiological actions of verapamil and D-600 in mammalian myocardium 0.3. Effects of optical isomers on transmembrane action potentials	Bayer, R; Kalusche, D; Kaufmann, R; Mannhold, R	290 (1975)	81	97
52	247	Comparative pharmacology of human beta-adrenergic receptor subtypes—characterization of stably transfected receptors in CHO cells	Hoffmann, C; Leitz, MR; Oberdorf-Maass, S; Lohse, MJ; Klotz, KN	369 (2004)	151	159
53	246	Differential-effects of clomipramine given locally or systemically on extracellular 5-hydroxytryptamine in raphe nuclei and frontal-cortex—an invivo brain microdialysis study	Adell, A; Artigas, F	343 (1991)	237	244
54	242	Possible subdivision of postsynaptic alpha-adrenoceptors mediating pressor-responses in the pithed rat	Timmermans, PBMWM; KWA, HY; Vanzwieten, PA	310 (1979)	189	193
55	241	Effect of diazepam on spinal-cord activities—possible sites and mechanisms of action	Polc, P; Möhler, H; Haefely, W	284 (1974)	319	337
56	241	Human drug metabolizing cytochrome P450 enzymes: properties and polymorphisms	Ingelman-Sundberg, M	369 (2004)	89	104
57	240	The anti-platelet and cardiovascular actions of a new carbacyclin derivative (ZK36374)—equipotent to PGI2 invitro	Schrör, K; Darius, H; Matzky, R; Ohlendorf, R	316 (1981)	252	255
58	239	Blockade of presynaptic alpha-receptors and of amine uptake in rat-brain by antidepressant mianserine	Baumann, PA; Maitre, L	300 (1977)	31	37
59	237	The use of tetrodotoxin for the characterization of drug-enhanced dopamine release in conscious rats studied by brain dialysis	Westerink, BHC; Tuntler, J; Damsma, G; Rollema, H; Devries, JB	336 (1987)	502	507
60	226	Inhibition of noradrenaline release in the rat-brain cortex via presynaptic H-3 receptors	Schlicker, E; Fink, K; Hinterthaner, M; Göthert, M	340 (1989)	633	638
61	223	Non-neuronal acetylcholine, a signalling molecule synthesized by surface cells of rat and man	Klapproth, H; Reinheimer, T; Metzen, J; Munch, M; Bittinger, F; Kirkpatrick, CJ; Hohle, KD; Schemann, M; Racke, K; Wessler, I	355 (1997)	515	523
62	220	Early desensitization of somato-dendritic 5-HT1A autoreceptors in rats treated with fluoxetine or paroxetine	Lepoul, E; Laaris, N; Doucet, E; Laporte, AM; Hamon, M; Lanfumey, L	352 (1995)	141	148
63	219	Meta-chlorophenylpiperazine—central serotonin agonist causing powerful anorexia in rats	Samanin, R; Mennini, T; Ferraris, A; Bendotti, C; Borsini, F; Garattini, S	308 (1979)	159	163
64	218	5-HT1A-receptors mediate stimulation of adenylate-cyclase in rat hippocampus	Markstein, R; Hoyer, D; Engel, G	333 (1986)	335	341
65	217	Capsaicin and nociception in the rat and mouse—possible role of substance-P	Gamse, R	320 (1982)	205	216
66	216	Comparison of the pharmacological characteristics of 5-HT1 and 5-HT2 binding-sites with those of serotonin autoreceptors which modulate serotonin release	Martin, Ll; Sanders-Bush, E	321 (1982)	165	170
67	216	5-hydroxytryptamine4 receptors mediate relaxation of the rat esophageal tunica muscularis mucosae	Baxter, GS; Craig, DA; Clarke, DE	343 (1991)	439	446
68	216	New selective ligands of human cloned melatonin MT1 and MT2 receptors	Audinot, V; Mailliet, F; Lahaye-Brasseur, C; Bonnaud, A; Le Gall, A; Amosse, C; Dromaint, S; Rodriguez, M; Nagel, N; Galizzi, JP; Malpaux, B; Guillaumet, G; Lesieur, D; Lefoulon, F; Renard, P; Delagrange, P; Boutin, JA	367 (2003)	553	561
69	213	Adenosine release from isolated fat-cells and its significance for effects of hormones on cyclic 3',5'-AMP levels and lipolysis	Schwabe, U; Ebert, R; Erbler, HC	276 (1973)	133	148
70	212	Concentration-dependent effects of tolbutamide, meglitinide, glipizide, glibenclamide and diazoxide on ATP-regulated K + currents in pancreatic B-cells	Zunkler, BJ; Lenzen, S; Manner, K; Panten, U; Trube, G	337 (1988)	225	230
71	212	Metabolism of risperidone to 9-hydroxyrisperidone by human cytochromes P450 2D6 and 3A4	Fang, J; Bourin, M; Baker, GB	359 (1999)	147	151
72	211	Inhibition of slow inward current by nifedipine in mammalian ventricular myocardium	Kohlhardt, M; Fleckenstein, A	298 (1977)	267	272
73	210	Autoradiographic characterization and localization of 5-HT(1D) compared to 5-HT(1B) binding-sites in rat-brain	Bruinvels, AT; Palacios, JM; Hoyer, D	347 (1993)	569	582
74	208	How reliable are G-protein-coupled receptor antibodies?	Michel, MC; Wieland, T; Tsujimoto, G	379 (2009)	385	388
75	207	Study of the contractile effect of 5-hydroxytryptamine (5-HT) in the isolated longitudinal muscle strip from guinea-pig ileum—evidence for 2 distinct release mechanisms	Buchheit, KH; Engel, G; Mutschler, E; Richardson, B	329 (1985)	36	41
76	206	Free fatty acids induce cholecystokinin secretion through GPR120	Tanaka, T; Katsuma, S; Adachi, T; Koshimizu, TA; Hirasawa, A; Tsujimoto, G	377 (2008)	523	527
77	206	Molecular pharmacology of somatostatin receptors	Hoyer, D; Lubbert, H; Bruns, C	350 (1994)	441	453
78	205	Presence and distribution of alpha-adrenoceptors in heart of various mammalian-species	Wagner, J; Brodde, Ee	302 (1978)	239	254
79	204	Evidence for common pharmacological properties of [H-3]5-hydroxytryptamine binding-sites, pre-synaptic 5-hydroxytryptamine autoreceptors in cns and inhibitory pre-synaptic 5-hydroxytryptamine receptors on sympathetic-nerves	Gillen, C; Haurand, M; Kobelt, DJ; Wnendt, S	362 (1983)	116	121
80	204	Affinity, potency and efficacy of tramadol and its metabolites at the cloned human mu-opioid receptor	Engel, G; Göthert, M; Müllerschweinitzer, E; Schlicker, E; Sistonen, L; Stadler, AA	324 (2000)	116	124
81	204	3 Classes of dopamine receptor (D-2, D-3, D-4) identified by binding-studies with apomorphine-H-3 and domperidone-H-3	Sokoloff, P; Martres, MP; Schwartz, JC	315 (1980)	89	102
82	202	Inotropic and electrophysiological actions of verapamil and D-600 in mammalian myocardium 0.1. Pattern of inotropic effects of racemic compounds	Bayer, R; Hennekes, R; Kaufmann, R; Mannhold, R	290 (1975)	49	68
83	201	Conjoint native and orthophthaldialdehyde-condensate assays for fluorimetric determination of 5-hydroxyindoles in brain	Atack, C; Lindqvist, M	279 (1973)	267	284
84	200	GTP-dependent inhibition of cardiac adenylate-cyclase by muscarinic cholinergic agonists	Jakobs, KH; Aktories, K; Schultz, G	310 (1979)	113	119
85	199	Presynaptic receptor systems on noradrenergic neurons of rabbit pulmonary-artery	Endo, T; Starke, K; Bangerter, A; Taube, HD	296 (1977)	229	247
86	199	Inhibitory effect of gamma.hydroxybutyric acid and gamma-aminobutyric acid on dopamine cells in substantia nigra	Anden, NE; Stock, G	279 (1973)	89	92
87	199	Endothelium-derived relaxant factor inhibits platelet activation	Busse, R; Lückhoff, A; Bassenge, E	336 (1987)	566	571
88	198	Die Noradrenalin-Abgabe aus dem isolierten Kaninchenherzen bei sympathischer Nervenreizung und ihre pharmakologische Beeinflussung	Hukovic, S; Muscholl, E	244 (1962)	81	&
89	197	Organic and inorganic calcium-antagonists reduce vasoconstriction in vivo mediated by postsynaptic alpha-2-adrenoceptors	Vanmeel, JCA; Dejonge, A; Kalkman, HO; Wilffert, B; Timmermans, PBMWM; Vanzwieten, PA	316 (1981)	288	293
90	197	Clonidine-induced inhibition of sympathetic-nerve activity—no indication for a central presynaptic or an indirect sympathomimetic mode of action	Haeusler, G	286 (1974)	97	111
91	197	Studies on mechanism of action of dantrolene sodium—skeletal-muscle relaxant	Ellis, KO; Carpenter, JF	275 (1972)	83	+
92	196	Selective stimulation of dopamine and noradrenaline autoreceptors by B-HT920 and B-HT933, respectively	Anden, NE; Golembiowskanikitin, K; Thornstrom, U	321 (1982)	100	104
93	196	Interaction of arylpiperazines with 5-HT1A, 5-HT1B, 5-HT1C and 5-HT1D receptors—do discriminatory 5-HTB receptor ligands exist	Schoeffter, P; Hoyer, D	339 (1989)	675	683
94	192	Benzodiazepine derivative and praziquantel—effects on musculature of schistosoma-mansoni and schistosoma-japonicum	Pax, R; Bennett, Jl; Fetterer, R	304 (1978)	309	315
95	192	Substance-P in the vagus nerve—immunochemical and immunohistochemical evidence for axoplasmic-transport	Gamse, R; Lembeck, F; Cuello, AC	306 (1979)	37	44
96	191	Tianeptine, a selective enhancer of serotonin uptake in rat-brain	Mennini, T; Mocaer, E; Garattini, S	336 (1987)	478	482
97	190	Involvement of cyclic-AMP in the direct inotropic action of amrinone—biochemical and functional evidence	Honerjäger, P; Schäfer-Korting, M; Reiter, M	318 (1981)	112	120
98	189	Clonidine antinociceptive activity—effects of drugs influencing central monoaminergic and cholinergic mechanisms in rat	Paalzow, G; Paalzow, L	292 (1976)	119	126
99	189	The significance of extracellular calcium for the release of dopamine, acetylcholine and amino-acids in conscious rats, evaluated by brain microdialysis	Westerink, BHC; Hofsteede, HM; Damsma, G; Devries, JB	337 (1988)	373	378
100	188	Renal effects of adenosine and their inhibition by theophylline in dogs	Osswald, H	288 (1975)	79	86

Data were retrieved from the Clarivate database on December 8th, 2021

As submissions to the journal steadily increased in the past years, so did the workload of the editorial board. The workload tripled from 2012 to 2020. However, the time until first decision decreased by 30% when comparing 2012 and 2021. Thus, the editorial board became more efficient. The fastest first decisions were recorded in 2021. It is possible that lockdowns related to the COVID-19 pandemic pushed editors and referees into the home office and gave them more time to edit and review, respectively, papers. Similar developments have also been noted for other journals (Palayew et al. 2020).

**Fig. 2:**
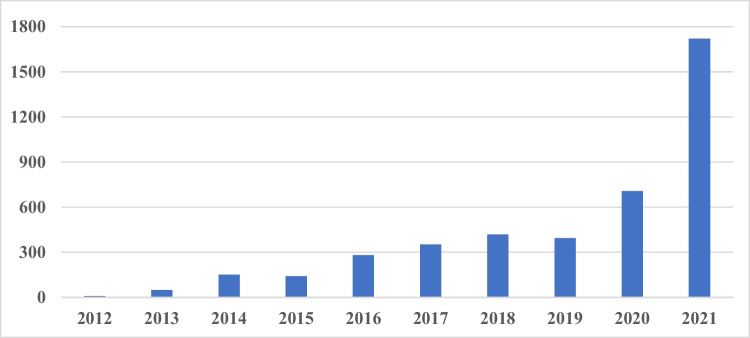
Social impact: total number of mentions (data available starting 2012)

In recent years, in addition to downloads, the presence in social media has become another parameter for assessing the relevance of a journal. We analyzed the Altmetrics score, which is a parameter representing an article’s attention by evaluating different sources, such as for example Tweets, blogs, and videos (https://www.altmetric.com/about-altmetrics/what-are-altmetrics/, last accessed June 9th, 2022). We analyzed the social impact for the years 2012–2021, viewing the total number of mentions. An exponential increase could be seen throughout the time span of ten years (Fig. 2). Whereas only nine mentions were recorded for the year 2012, there were 706 mentions in 2020 and 1720 mentions made in 2021. Especially a significant increase in tweets about the journal could be shown in 2021. This is most likely due to the efforts of the journal to fight the paper mills (Seifert 2021).

In addition, *Naunyn–Schmiedeberg’s Archives of Pharmacology* is a journal which not only publishes positive results but encourages authors to publish solid “negative” results (Seifert 2016). Therefore, authors can publish “negative” data on controversial issues in the journal which are then discussed in social media.

### Analysis of the top contributors to the journal

**Fig. 3:**
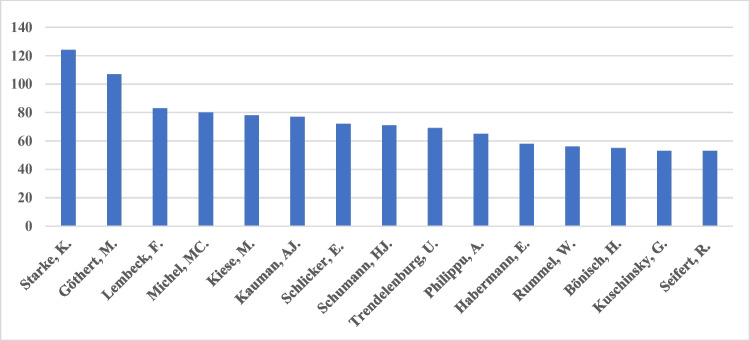
Top-15 contributors to the journal

We investigated the journal’s top-15 contributing authors from 1947 to 2020 (Fig. 3). All these authors published more than 40 papers in the journal. Klaus Starke (Freiburg) is leading the top-15 group with more than 120 articles, followed by Manfred Göthert (Bonn) with more than 100 publications in the journal. Starke mostly published on the adrenergic system, whereas Göthert focused on the serotonergic system (https://pubmed.ncbi.nlm.nih.gov, last accessed June 9th, 2022). Remarkably, these two top contributors published more than 30% of all their research papers in *Naunyn–Schmiedeberg’s Archives of Pharmacology* and held chair positions of large influential academic pharmacology departments. These two scientists had a major impact on the major research topics of the journal for decades and the contribution of cities to the journal (see discussion below). Starke and Göthert also provided service to the journal as editors (Starke 1998; Bönisch et al. 2021).

Starke and Göthert are followed, with a clear margin, by a group of pharmacologists working in related fields (e.g., in alphabetical order, Bönisch, Kaumann, Michel, Philippu, Schlicker, Schümann, Seifert, Trendelenburg). Other fields of pharmacology such as cardiovascular pharmacology (Kuschinsky), pharmacokinetics (Kiese), bacterial toxins (Habermann), substance P (Lembeck), and transport (Rummel) are covered as well (Philippu, 2004–2021).

### Analysis of 100 most-cited articles

We analyzed the 100 most-cited articles published in *Naunyn–Schmiedeberg’s Archives of Pharmacology* using the Clarivate database. The database covers papers from 1947 onwards. We analyzed papers from 1947 to 2020. Table 1 presents the 100 most-cited articles. The number of citations for the highest cited publication is 1157. We noted that the most represented document types were original articles (83%). Review articles accounted for just 11% of the top papers, supporting the view that *Naunyn–Schmiedeberg’s Archives of Pharmacology* is a classic original research journal (see discussion above). Few proceeding papers, notes, and editorial material are among the top-100 papers (Fig. S1).

**Fig. 4:**
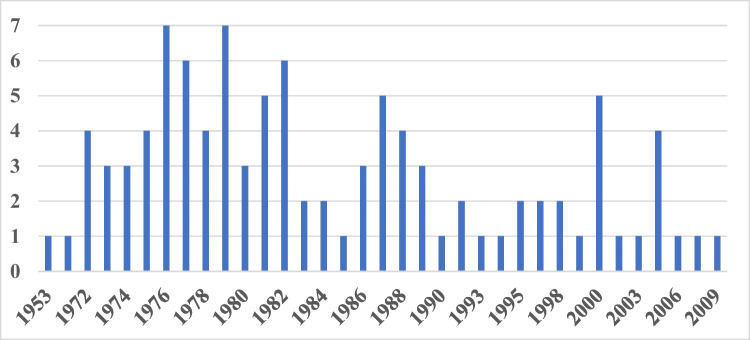
100 most-cited articles: articles per year

When analyzing the publication years, we noticed that most of the most-cited articles were published between 1977 and 1979 (Fig. 4), i.e., shortly after English was introduced as official language (Starke 1998; see discussion below). These findings indicate that the switch to English greatly broadened international reception of research at that time. Only two papers (ranks 9 and 87) are in German language. The most recent top-100 paper is from the year 2009. Thus, the late 1970s were a particularly successful period for the journal. Back then, the impact factor did not play a role in assessing the perceived quality of a journal.

**Fig. 5:**
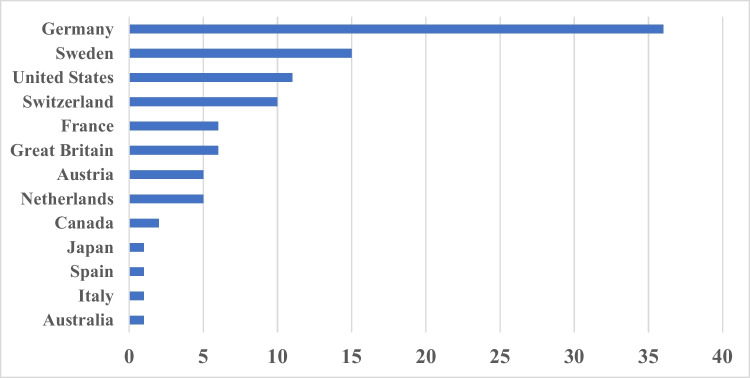
100 most-cited articles: countries

Furthermore, we investigated the 100 most-cited articles for their geographical origin focusing on country and city of origin. Germany is the leading country with 36 papers, followed by Sweden (15), the USA (11), and Switzerland (10) (Fig. 5). All the Swiss papers came from Basel, making it the leading city in terms of contributing highly cited papers to *Naunyn–Schmiedeberg’s Archives of Pharmacology* (Fig. S2). Basel is ranked before Freiburg, Gothenburg, and Essen, Graz, Stockholm, Bonn, Wurzburg, Amsterdam, and Cambridge. Most notably, the Basel papers originated in the pharmaceutical industry, and not in academia. This is a unique situation in the sense that pharmaceutical industry, in a certain period of history, made substantial contributions to basic pharmacology and published the results in an academic journal. For the first three highest ranked cities, the adrenergic and serotonergic system constituted the major research topics.

**Fig. 6:**
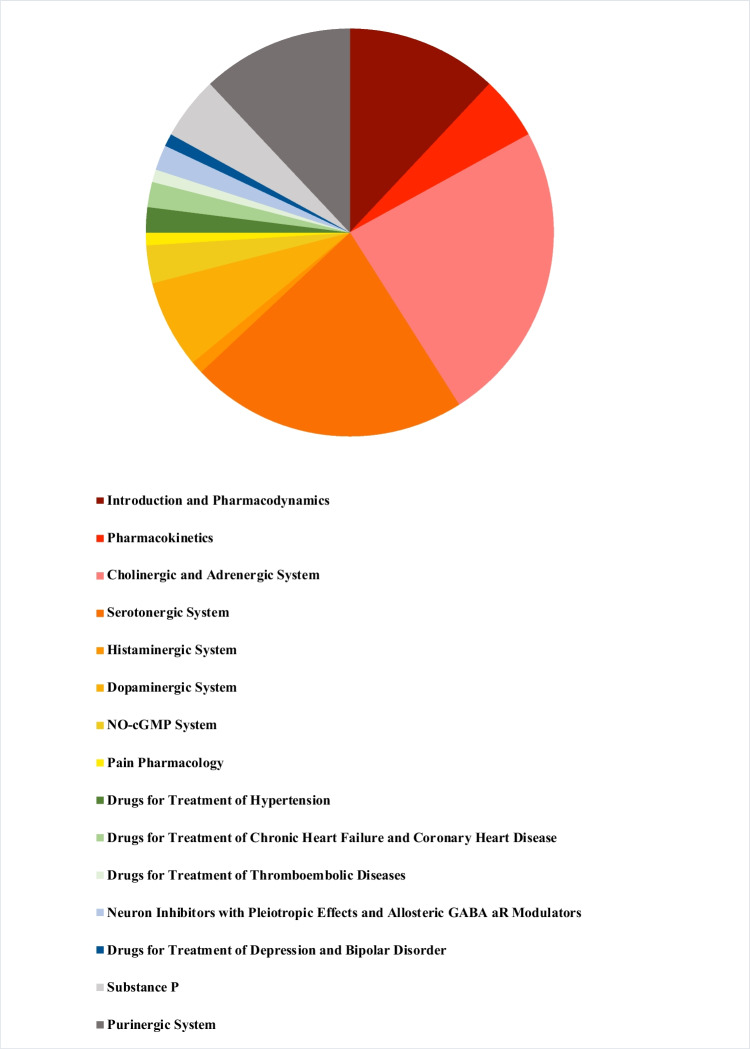
100 most-cited articles: topics by chapters of “Basic Knowledge of Pharmacology”

We assigned the titles of the 100 most-cited papers to chapters of the textbook “Basic Knowledge of Pharmacology” (Seifert 2019) which is broadly used in pharmacology classes (https://link.springer.com/book/10.1007/978-3-030-18899-3; last accessed August 4th 2022). “Cholinergic and Adrenergic System” was the most common topic (24%), followed by serotonergic system (22%). Neurotransmitter papers (including histaminergic, dopaminergic purinergic, and peptidergic system) amounted to > 50% of the most highly cited papers (Fig. 6). Papers assigned to “Introduction and Pharmacodynamics” contributed to > 10% to the highly cited papers. Thus, from the perspective of the most-cited papers, *Naunyn–Schmiedeberg’s Archives of Pharmacology* can be viewed as neurotransmitter and pharmacodynamics journal.

When looking at times cited, one article significantly stands out, having almost 1200 citations (Table 1 and Fig. S3). This paper is entitled “Substance-P as neurogenic mediator of antidromic vasodilation and neurogenic plasma extravasation.” It was published by F. Lembeck and P. Holzer in 1979 and is an original article. It was submitted from the University of Graz in Austria. Since 2013, 12 accesses to the article could be registered in Clarivate. This clearly shows that even very old original research is still relevant and being accessed more than 30–40 years after publication. The contribution of such citation classics is, however, not captured in the popular and predominant impact factor that covers only the past 2 years but not the long-term impact of research.

### Analysis of zero-cited articles

We also regarded zero-cited articles into our analysis, trying to determine trends making an article less “successful” or at least less requested. We included articles that listed zero citations from 2015 to 2022, as of April 2022. A total of 165 publications could be registered with zero citations, 113 of those being “original articles” and 11 “review articles “Retractions,” “corrections,” “editorial material,” and “expression of concern” are also included as types of articles (Fig. S4). Overall, this pattern is similar to the distribution of paper types found for the most-cited articles (Fig. S6). Most importantly, even review articles are well represented in the zero-cited category. This came as a surprise as general dogma holds that review articles get more cited than original articles. Thus, at least for *Naunyn–Schmiedeberg’s Archives of Pharmacology*, review articles may be less important for international recognition than is intuitively assumed.

**Fig. 7:**
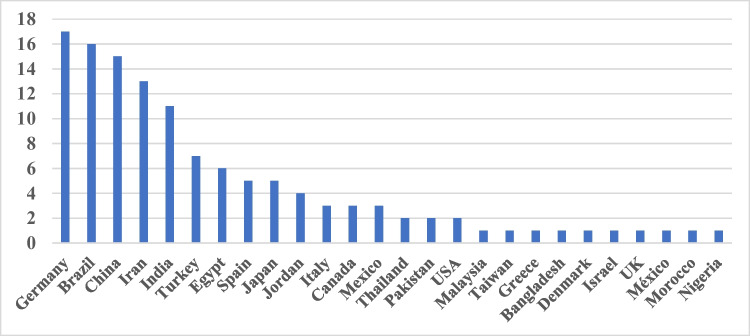
Zero-cited articles: countries

Most zero-cited articles came from Germany (17), followed by Brazil (16), China (13), and Iran (13) (Fig. 7). These data fit well to the countries of origin of current papers in the journal with China, Egypt, Brazil, and Iran leading the field (see discussion below). Thus, there is no bias in citation of papers from China, Egypt, Brazil, and Iran. Rather, the opposite is true with papers from Germany being not cited over proportionally. If a paper is deemed to be relevant, it will be cited, regardless of origin.

Topics of zero-cited papers were assigned to chapters of “Basic Knowledge of Pharmacology” (Seifert 2019). “Drugs for the Treatment of Malignant Tumor Diseases” (24), “Immunopharmacology” (16) and “Pharmacodynamics” (25) were among the most common topics (Fig. S5), fitting quite well to the pattern of contemporary research topics (see discussion below). Papers on pharmacodynamics were overrepresented among the zero-cited papers. Perhaps, currently, *Naunyn–Schmiedeberg’s Archives of Pharmacology* is perceived more as a disease-oriented journal than a basic molecular pharmacology journal.

### Analysis of meta-data

We studied the meta-data of *Naunyn–Schmiedeberg’s Archives of Pharmacology* in different dimensions to present a long-term analysis of the journal. Due to multiple unforeseen obstacles, such as wrongly appointed or missing data, we had to limit our analyses to different times for each parameter. Thus, we focused on the years 1947–1976 for the publication language analysis, on the years 1990–2020 for the geographical analysis, and on the years 1970, 1980, 1990, 2000, 2010, and 2020 for the analysis of research topics.

**Fig. 8:**
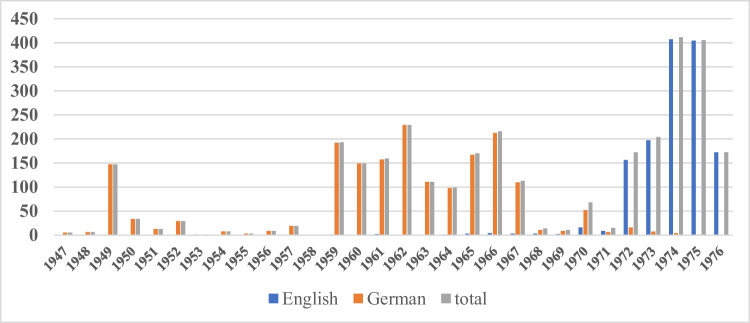
Meta-data: publication languages (1947–1976)

After World War II, publications in the journal started again in 1947 with a sharp peak in 1949 (Fig. 8 and Fig. S6). This peak reflects publication of research that was performed during the war but could not be published because of the disruption of civil infrastructure and communication. At that time, virtually, all papers were published in German language. In the following 10 years, few papers were published in *Naunyn–Schmiedeberg’s Archives of Pharmacology*. This reflects the fact that badly destroyed research institutes had to be rebuilt and new research projects had to be started from scratch (Philippu, 2004–2021). From 1959 to 1966, *Naunyn–Schmiedeberg’s Archives of Pharmacology* published up to 210 papers per year, virtually all in German and reflecting recovery of pharmacological research in Germany after World War II. But in 1968, a situation like in 1950 emerged, and only very few papers were published in the following 2 years. This is probably due to the fact that at this time, pharmacologists from Germany wished to regain international recognition after World War II and started to publish in journals publishing in English language (Philippu 2004–2021). This situation, bringing the journal close to the brink of disappearance (Starke 1998), forced the editors Fred Lembeck and Ullrich Trendelenburg to abandon German language and switch to English language. Part of the decision was to rename the journal to *Naunyn–Schmiedeberg’s Archives of Pharmacology*, the name being valid until today. This decision turned out to very prudent. In the year 1972, publication numbers of the journal had recovered and reached an ever high of more than 400 papers per year in 1974 and 1975. Thus, the transition from German to English language was forced by existential crisis, implemented abruptly and successful. Figure 8 also highlights the huge damage Germany had inflicted itself on pharmacological research from 1933 to 1945. It took more than 25 years after the war to rebuild international recognition of German pharmacology. The results of the research were then published in English.

**Fig. 9:**
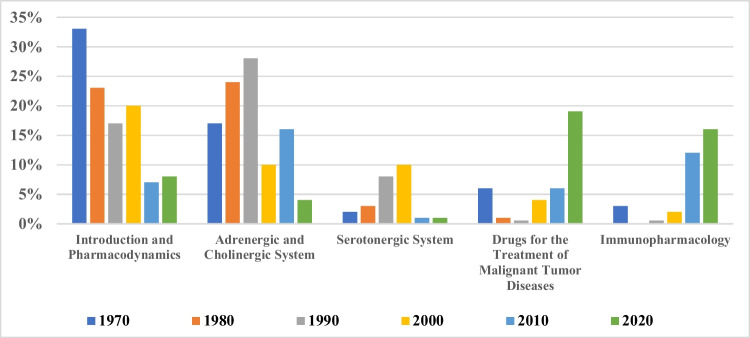
Meta-data: development of most used topics (1970–2020)

We used the years 1970, 1980, 1990, 2000, 2010, and 2020 for a thematical study of the journal’s publications throughout the decades. Each year is set to represent a decade. For a unified approach, we assigned a color to each chapter of “Basic Knowledge of Pharmacology” (Seifert 2019) (Figs. S7, S8, S9, S10, S11, and S12). As done before, additional categories were added, namely “Purinergic System,” “Editorial Analysis,” “Substance P,” and “Drugs for Treatment of Parasites´ Infections.” In 1970, the most represented chapter was “Introduction and Pharmacodynamics” (Fig. 9; Fig. S7); in 2020, it was “Drugs for the Treatment of Malignant Tumor Diseases” (Fig. S12). “Immunopharmacology” also rose to become the second most represented category in 2020. The contribution of neurotransmitter pharmacology to *Naunyn–Schmiedeberg’s Archives of Pharmacology* declined substantially over the decades, a major reason being that top contributors to the field retired or even passed away (Bönisch et al. 2021). The importance of *Naunyn–Schmiedeberg’s Archives of Pharmacology* as a general mechanistic journal dedicated to molecular mechanisms of drug action also declined over the decades (“Introduction and Pharmacodynamics”).

The yearly share of our additionally added categories shows differences, with not every category always being represented (Figs. S7, S8, S9, S10, S11, S12, S13, S14, S15, S16, and S17). Significantly “Purinergic System” stands out, being represented by 10 articles in 1990 and by 17 articles in 2000 (Figs. S9 and S10). 2020 is the only year, where the category “Drugs for Treatment of Viral Infections” was assigned (Fig. S12). Five articles can be assigned to this topic in 2020, all discussing COVID-19. This is the response to the pandemic, showing the timeliness of the journal and its constant development in presenting topics of high relevance. The relevance of the topic can also be seen by the fact that a COVID-19 paper was the most often downloaded and cited paper in 2020 (Rizzo 2020; https://link.springer.com/article/10.1007/s00210-020-01902-5; last accessed August 4th, 2022).

The number of textbook chapters represented in *Naunyn–Schmiedeberg’s Archives of Pharmacology* steadily increased over the past 50 years. While 17 categories were represented in 1970, we recorded 27 categories in 2010 and 30 categories in 2020 (Figs. S7, S11, and S12). Thus, the diversity of research topics in *Naunyn–Schmiedeberg’s Archives of Pharmacology* increased and the number of research fields covered in the journal almost doubled.

**Fig. 10:**
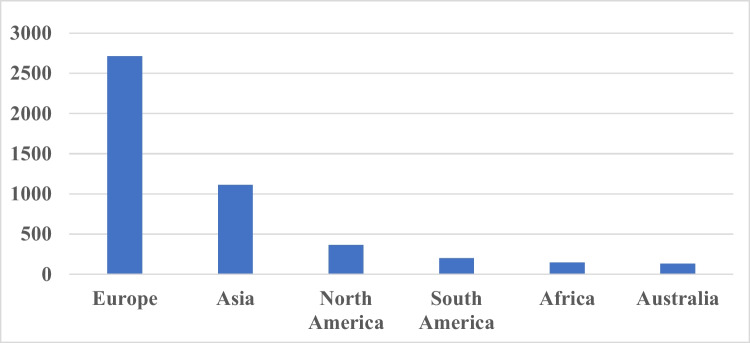
Meta-data: publications by continent (1990–2020)

For a geographical overview, we first analyzed the number of publications by continent. Europe is the leading continent, being the origin to more than 2500 publications from 1990 to 2020 (Fig. 10). Europe is followed by Asia (1,110), North America (363), South America (200), Africa (145), and Australia (131). Thus, *Naunyn–Schmiedeberg’s Archives of Pharmacology* is deeply rooted in Europe but can be viewed as a globally recognized journal if all other continents are added up.

When analyzing the top-20 contributing countries, ten European countries can be named; these countries being Germany, Italy, the UK, Spain, France, the Netherlands, Sweden, Poland, Austria, and Switzerland. Combined, these countries make up close to 90% of total European publications (Fig. S13). The European lead is mainly due to German publications, as Germany is the country with the most publications (1163). Germany is followed by non-European countries, i.e., Japan and the USA. China, Brazil, Australia, Egypt, India, Taiwan, and Canada complete the top 20. Historically, Japan and Germany always had close research connections, and this also applies to pharmacology (Philippu, 2004–2021; Hattori et al. 2022).

Interestingly, when viewing publications over time, dramatic differences in publication numbers become evident. Despite Germany being the leading country from 1990 to 2016, its numbers decreased from > 70 publications in 1995 to an all-time low of mere 14 published papers in 2016 (Fig. S14). In other words, within just 20 years, *Naunyn–Schmiedeberg’s Archives of Pharmacology* lost about 80% of its historical paper contribution from Germany, although it is the official journal of the German Society for Experimental and Clinical Pharmacology and Toxicology (https://www.springer.com/journal/210/; last accessed August 4th, 2022). These numbers show that for German pharmacologists, ties to the official society journals are loose from the author perspective. However, in the Editorial Board where the Editor-in-Chief decides on editor appointment, representation of German Pharmacologists is still very substantial, i.e., 80% of all editors are members of the German Society for Experimental and Clinical Pharmacology and Toxicology (https://www.springer.com/journal/210/editors; last accessed August 4th, 2022).

The analysis of the top-10 contributing countries in a time series shows that annual fluctuations affected each country (Fig. S14). Japan shows significant spikes in the years 2002 and 2007–2009.

**Fig. 11:**
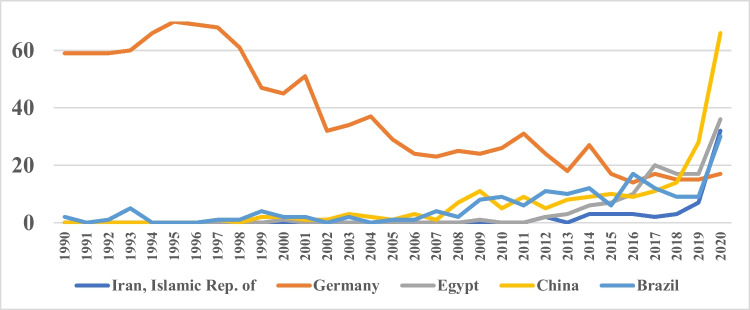
Meta-data: publications from Germany, Egypt, China, Brazil, and Iran (1990–2020)

Paradigmatic trends can be seen when viewing Egypt, Brazil, Iran, China, and Germany over time. These countries have been selected showing contrary developments and the global spread of pharmacological research (Fig. 11). In 2020, 66 papers were from China, with the first Chinese paper published in 1997. Thus, just within two decades, China has become the major contributor to *Naunyn–Schmiedeberg’s Archives of Pharmacology*. In 2020, Egypt had 36 publications, followed by Iran with 32 papers and Brazil with 30 papers, all far surpassing Germany with just 17 publications. In 2020, Germany contributed less than 8% to all papers published in *Naunyn–Schmiedeberg’s Archives of Pharmacology*, an all-time low.

The major driving force for the dramatic decline of publications from Germany in *Naunyn–Schmiedeberg’s Archives of Pharmacology* is certainly the *zeitgeist* placing high emphasis on publications in high-impact factor journals for successful academic careers in pharmacology (Zehetbauer et al. 2022 and discussion above). It is less clear for which reasons *Naunyn–Schmiedeberg’s Archives of Pharmacology* is so popular among pharmacologists from China, Egypt, Iran, and Brazil. No systematic data are available. Anecdotal evidence suggests that the quality and fairness of the peer review process as well as the quality of the editorial board play a major role in the popularity of the journal in some countries.

Analyzing the top-20 contributing cities, Freiburg and Bonn are leading with a clear margin to Mainz (Fig. S15). The high numbers for Freiburg are due to Klaus Starke, and the high numbers of Bonn are due to Manfred Göthert. Basel (Switzerland) is fourth. Tokyo, Taipei, Melbourne, Hanover, Heidelberg, and Essen complete the top 10. Tubingen, Frankfurt am Main, Stockholm, Porto, Berlin, Florence, Cairo, Amsterdam, Wurzburg, Paris, and Cambridge are the following most publishing cities, making the top-20 count complete. Thus, 50% of top-20 cities are from Germany, and the remainder of the cities is from other European countries, Asia, Africa, and Australia. Thus, *Naunyn–Schmiedeberg’s Archives of Pharmacology* has a broad international representation. The lack of representation of the USA in the top-20 contributing cities is probably since there are multiple cities in the USA contributing to the journal and not, in contrast to other countries, a dominating city (e.g., Basel (Switzerland); Tokyo (Japan); Taipeh (Taiwan)).

**Fig. 12:**
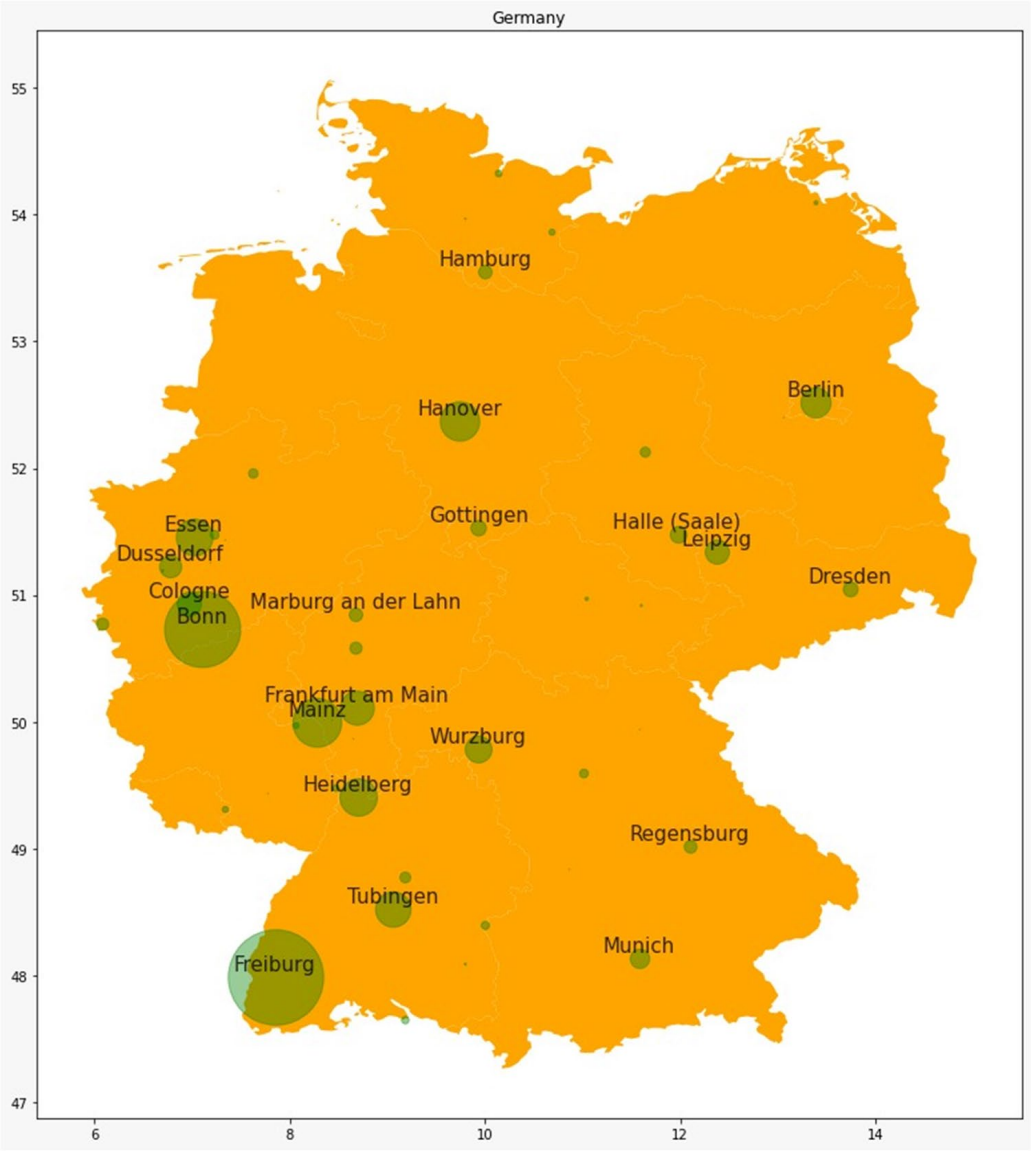
Geographical map of germany; hotspot publication cities (1990–2020)

The German cities with the most substantial contribution to *Naunyn–Schmiedeberg’s Archives of Pharmacology* are located in the West and South-West parts of Germany (Fig. 12). This reflects the fact that in this part of Germany (the old Federal Republic of Germany, FRG, West-Germany), after World War II, pharmacological institutes were rebuild generously and could readily publish in *Naunyn–Schmiedeberg’s Archives of Pharmacology* (Starke 1998). In contrast, in the Eastern part of Germany (the earlier German Democratic Republic, GDR, East-Germany), rebuilding of pharmacological institutes was much slower, and access to *Naunyn–Schmiedeberg’s Archives of Pharmacology* was very limited (Starke, 1998; Philippu 2004–2021). Although Fig. 12 analyzes the publication record in the 30 years after the reunification of Germany, nonetheless, the delay in rebuilding hotspots in pharmacological research in the East is very evident. The only hotspot in the East is Berlin, the reasons for its prominence being the fact that the western part of Berlin benefited from financial support from the FRG during the German division and that the city hosts multiple research institutions with pharmacological institutes (Philippu 2004–2021). Other notable hotspots are Hanover (North-Central); Essen (West) and Regensburg (South-East). Universities in these cities were newly founded in the 1960s–1970s with the expansion of higher education for broad segments of the German population. These new pharmacological institutes rapidly adopted *Naunyn–Schmiedeberg’s Archives of Pharmacology* as a venue for publication. Thus, the popularity of *Naunyn–Schmiedeberg’s Archives of Pharmacology* as a publication outlet is not limited to universities with a long history of pharmacology departments such as Freiburg, Bonn, and Mainz. These data show that a traditional journal can be very attractive for modern institutions.

## Conclusions and future perspectives

In 2023, *Naunyn–Schmiedeberg’s Archives of Pharmacology* is celebrating its 150th anniversary, and the journal is in an excellent condition, despite having encountered multiple serious challenges such as the self-inflicted annihilation of German pharmacology in World War II, the necessity to switch from German to English language in the 1970s, the retirement of leading contributors to the journal in the 2000s, the dramatic decline in papers from Germany during the past 25 years, and the paper mill crisis in 2020 (Seifert 2021; Sabel and Seifert 2021; van der Heyden 2021).

This paper shows how the journal has successfully adapted and reacted to these challenges and evolved from a German neurotransmitter journal into an international pharmacological journal with authors from all continents and representing a broad spectrum of topics with an emphasis on tumor pharmacology and immunopharmacology. The paper also shows how eminent scientists have shaped the journal in terms of research topics and geographical location. The journal has been strongly influenced by German history, specifically World War II and the division of the country following the war. Effects of these periods, going back decades, are still visible.

Writing and publishing scientific papers are cultural acts that are strongly influenced financial and career reward systems. The strong impact of such overwhelming *zeitgeist* attitudes is most evident with the dramatic decrease of publications from Germany in *Naunyn–Schmiedeberg’s Archives of Pharmacology* over the past 25 years. This unfortunate development could have brought the journal, again, to the brink of extinction, but particularly, during the past 5–10 years, submissions from other countries compensated for the loss in submissions from Germany. This resulted in large shifts in the topics covered by *Naunyn–Schmiedeberg’s Archives of Pharmacology.* We have no evidence that research from countries with emerging pharmacological research such as China, Egypt, Iran, and Brazil is undercited. Traditionally, *Naunyn–Schmiedeberg’s Archives of Pharmacology* has been predominantly an original research paper journal. In this context, it is interesting to note that even reviews may go uncited, suggesting that increasing the number of reviews in a journal may not automatically result in higher citation rates and a higher impact factor.

This paper also provides interesting insights into the life of scientists, i.e., in which month they read scientific papers and how they coped with the COVID-19 pandemic. The paper documents the successful transition of the journal from a print journal to an online journal and the exponentially progressing representation of the journal in social media. This paper provides evidence for the notion that even very old research papers are cited in current times. Finally, the paper points to societal issues, i.e., the non-representation of women among the top-contributing authors.

What will be the future development of the journal? *Naunyn–Schmiedeberg’s Archives of Pharmacology* is known for its fair and constructive review process (Starke 1998; Seifert 2016). The journal has a “science first” policy, allowing flexible publication formats and no incentives to artificially increase its impact factor. Based on the diversification of research topics that took place over the past decades, the journal will continue to welcome publications from every field of pharmacology, even “small” fields that may yield only few citations. The present paper has shown that it may take decades to assess the true scientific value of a paper.

We anticipate that the journal will strengthen its position in the fields of tumor pharmacology and immunopharmacology. Based on the diverse research topics presented in the journal and a broad global authorship, we predict a bright future for the journal. When Starke wrote his review in 1998, he predicted that the solid submission basis from Germany would be an asset to the journal. This turned out not to be the case, but nonetheless, the journal survived.

*Naunyn–Schmiedeberg’s Archives of Pharmacology*, although being a traditional pharmacology journal, will be up to date, e.g., as with the implementation of a mechanistic drug class nomenclature (Seifert and Schirmer 2021; Seifert and Alexander 2022). The journal will also welcome high-quality studies on chemically defined natural compounds (Merfort et al. 2017) and biologically well-defined probiotics (Neumann and Seifert 2021). However, the journal will not publish purely computational studies without pharmacological experiments (https://www.springer.com/journal/210/submission-guidelines?IFA#Instructions%20for%20Authors_Important%20Submission%20Policy, last accessed August 4th, 2022) and not pharmacological studies dealing only with crude biological extracts (Merfort et al. 2017). Lastly, the journal is aware of its societal and historical responsibility and will welcome papers connecting pharmacology and society (Zehetbauer et al. 2022; Ellerbeck and Seifert 2022) or pharmacology and history (Löffelholz 2011; Pohar and Hansson 2021; Philippu et al. 2022). With regret, we noted that pharmacodynamics topics are much less prominently presented in *Naunyn–Schmiedeberg’s Archives of Pharmacology* than they used to be, but this may also be a result of *zeitgeist* placing emphasis on applied disease-oriented research which is strongly represented in the journal. Nonetheless, pharmacodynamics papers are explicitly welcome.

Taken together, we hope that this article, putting together some numerical parameters of *Naunyn–Schmiedeberg’s Archives of Pharmacology*, revealed some new insights into the history, inner workings, and scientific development of the journal and could convince the reader to consider the journal for the next research paper and/or review.

## Supplementary Information

Below is the link to the electronic supplementary material.

Supplementary file1 (PDF 481 KB)

## Data Availability

All source data for this study are available upon reasonable request from the authors.
