# Correction to: Bibliometric development of Naunyn–Schmiedeberg’s Archives of Pharmacology

**DOI:** 10.1007/s00210-022-02335-y

**Published:** 2022-12-14

**Authors:** Leah B. Dats, Florentin von Haugwitz, Roland Seifert

**Affiliations:** 1 Institute of Pharmacology, Hannover Medical School, Carl‑Neuberg‑Str. 1, 30625 Hannover, Germany; 2 Nova School of Business and Economics Carcavelos Campus, Rua da Holanda, no. 1, 2775‑405 Carcavelos, Portugal

**Correction to: Naunyn–Schmiedeberg’s Archives of Pharmacology**

10.1007/s00210-022-02307-2

Due to an oversight by the Publisher during the publication process, the paper has been published prematurely. The author requested to see the revised proof before proceeding to the online publication as there were additional errors spotted. Received the additional correction from the author and the paper has now been updated.

The Publisher apologizes for this error.

