# Supplementary material for: Bibliometric development of Naunyn–Schmiedeberg’s Archives of Pharmacology
Source: Naunyn Schmiedebergs Arch Pharmacol. 2022 Oct 25;396(1):43–61. doi: 10.1007/s00210-022-02307-2 (PMC9592544; doi:10.1007/s00210-022-02307-2)
Supplement: Supplementary file 1 — Supplementary file1 (PDF 481 KB) [file 210_2022_2307_MOESM1_ESM.pdf]

Supplemental Figures S1-S15

Bibliometric development of

Naunyn-Schmiedeberg's Archives of Pharmacology

Leah Dats, Florentin von Haugwitz, Roland Seifert

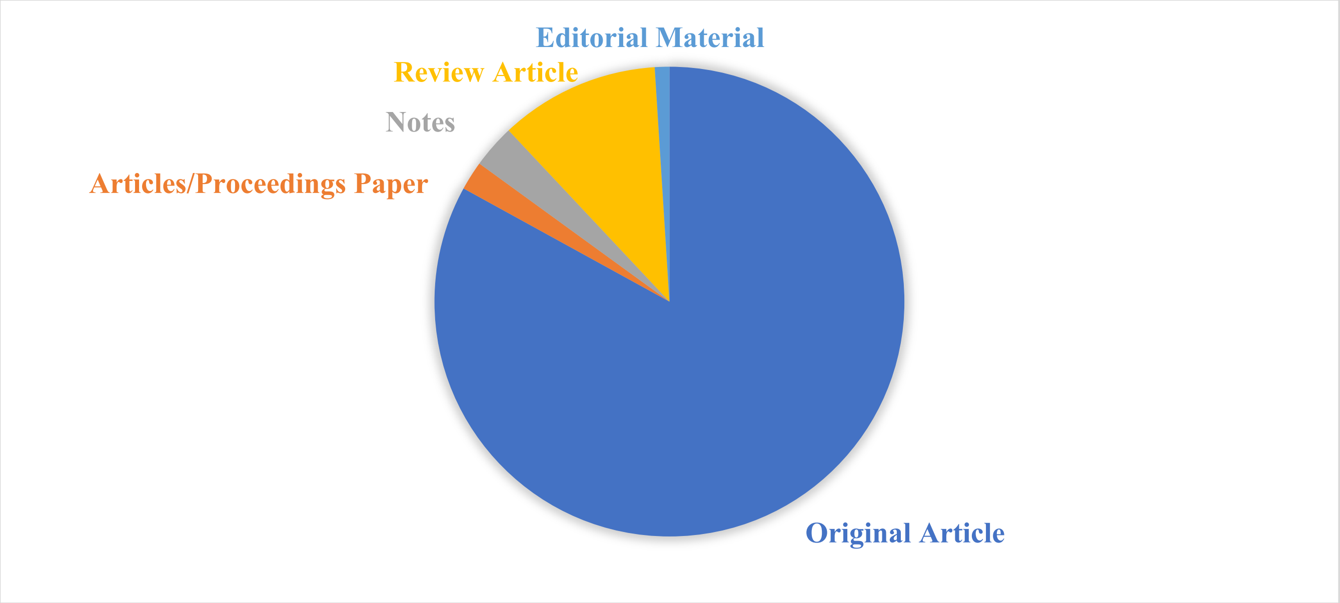

Fig. S1 100 Most cited articles: Document type

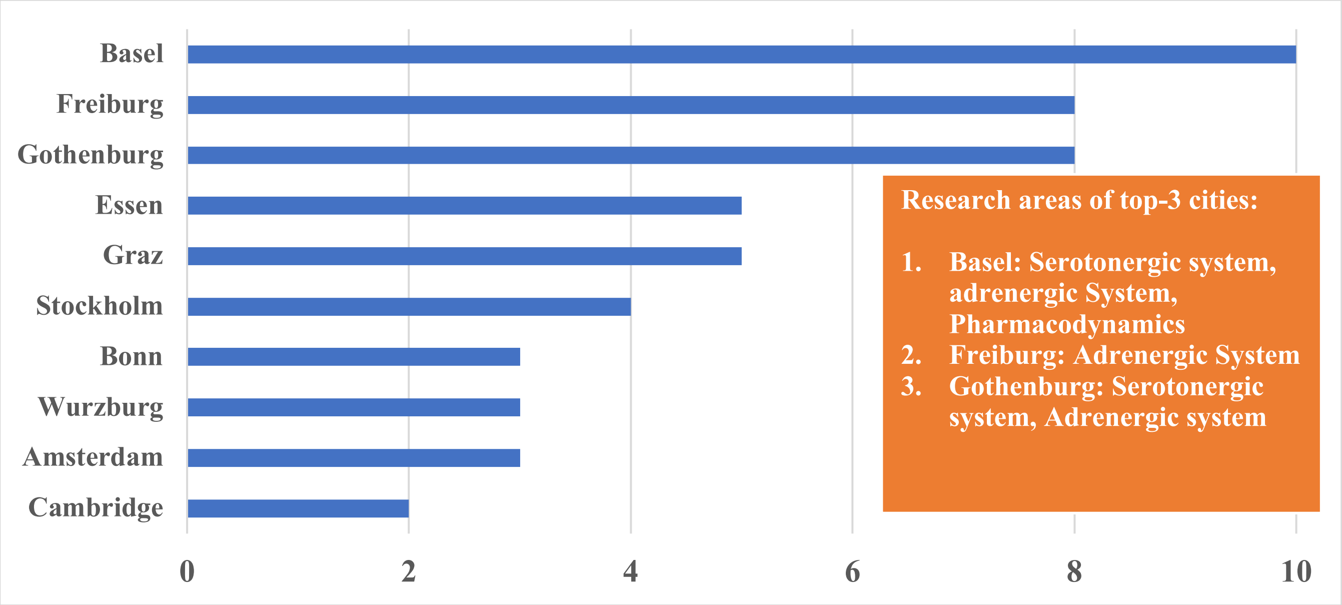

Fig. S2 The 100 most cited articles: Top-10 cities

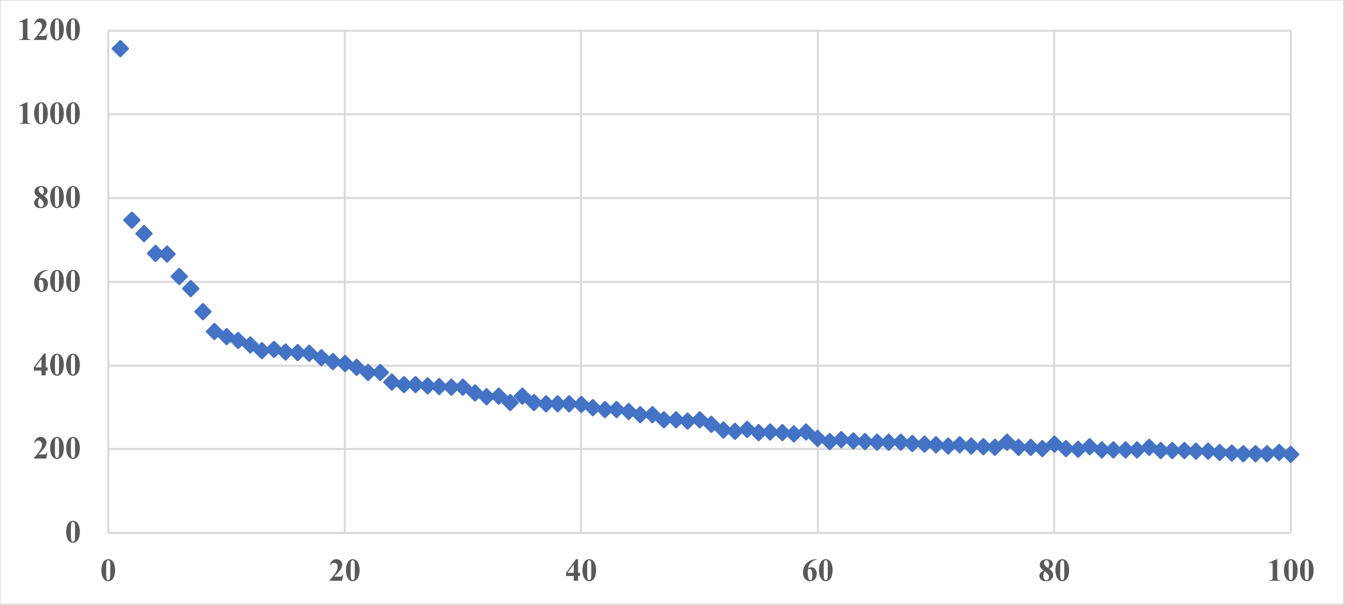

Fig. S3 The 100 most cited articles: Times cited, all databases (as of December 8th 2021)

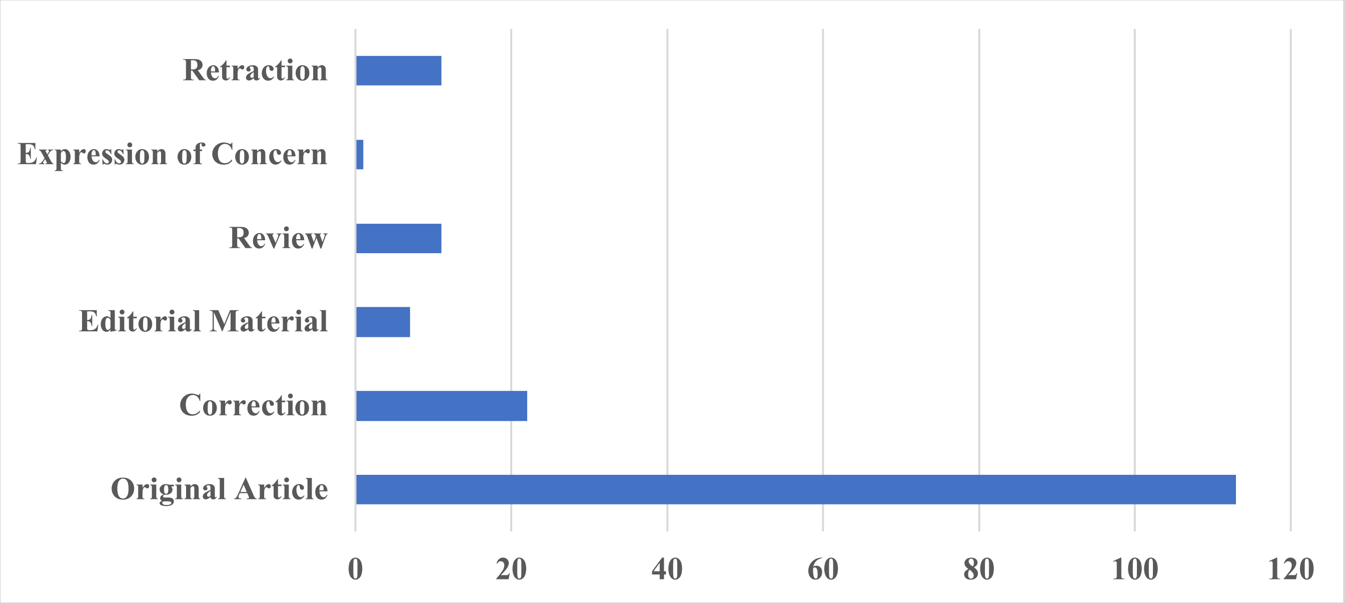

Fig. S4 Zero cited articles: Article types (2015-2022, as of April 2022)

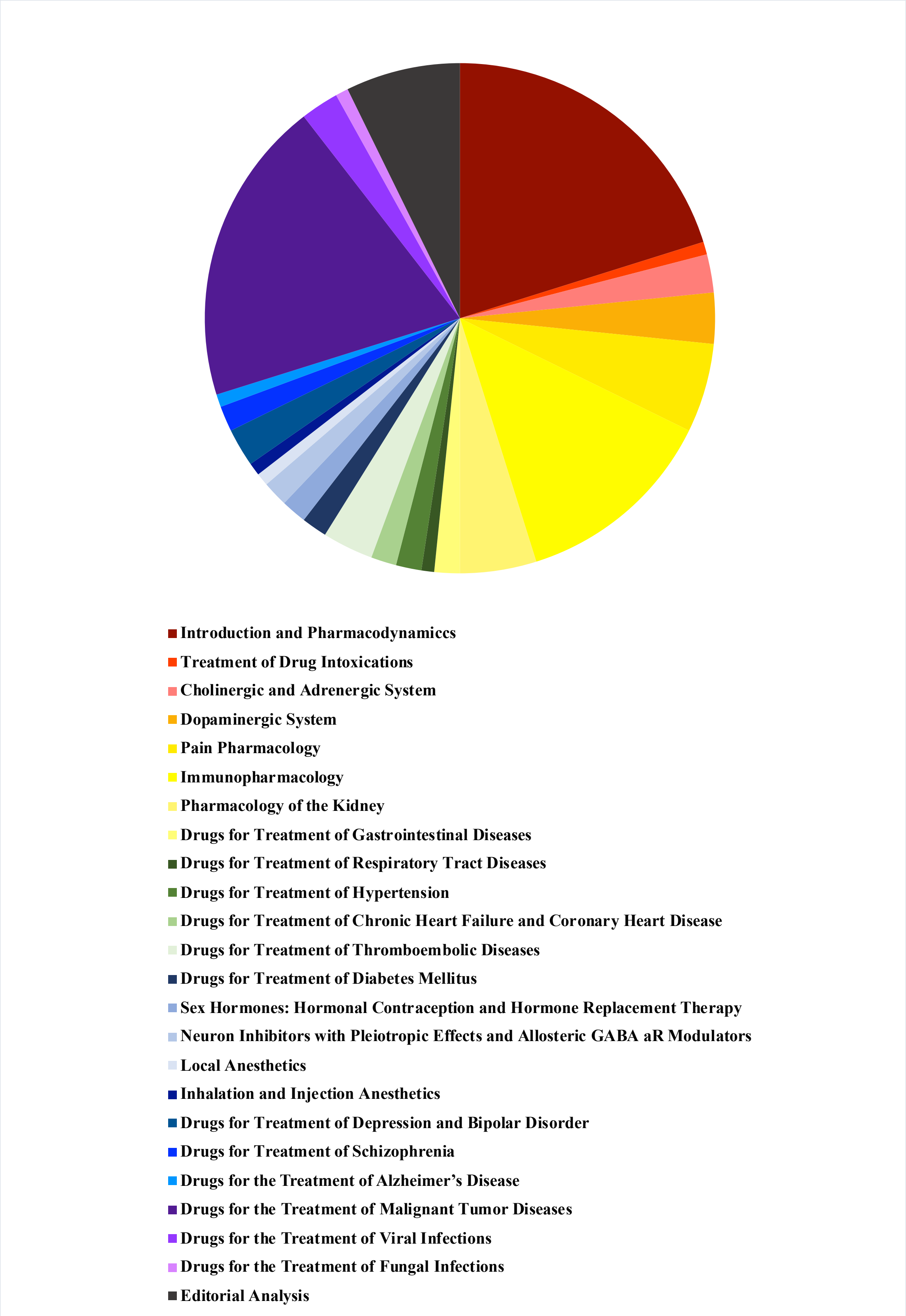

Fig. S5 Zero cited articles: Topics by chapters of "Basic Knowledge of Pharmacology "

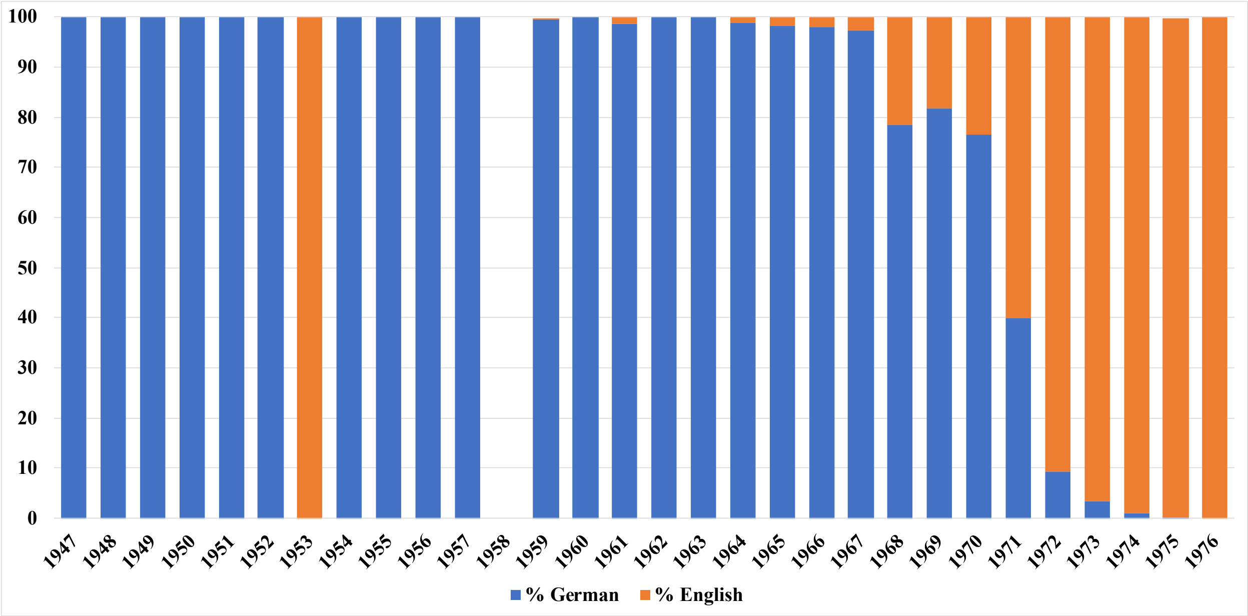

Fig. S6 Meta-data: Publication languages: Percentages for the years 1947-1976

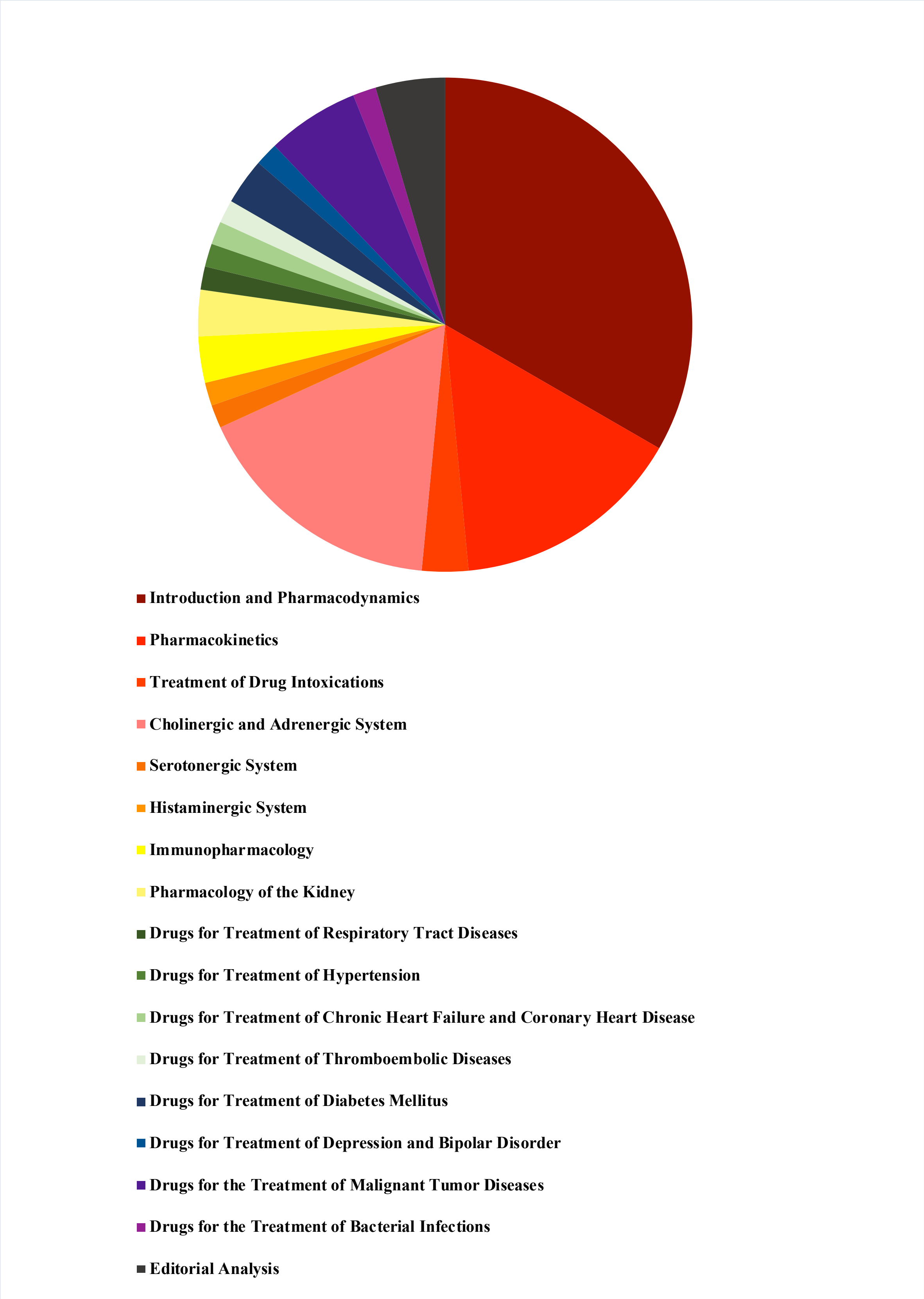

Fig. S7 Meta-data: 1970 Topics by chapters of "Basic Knowledge of Pharmacology"

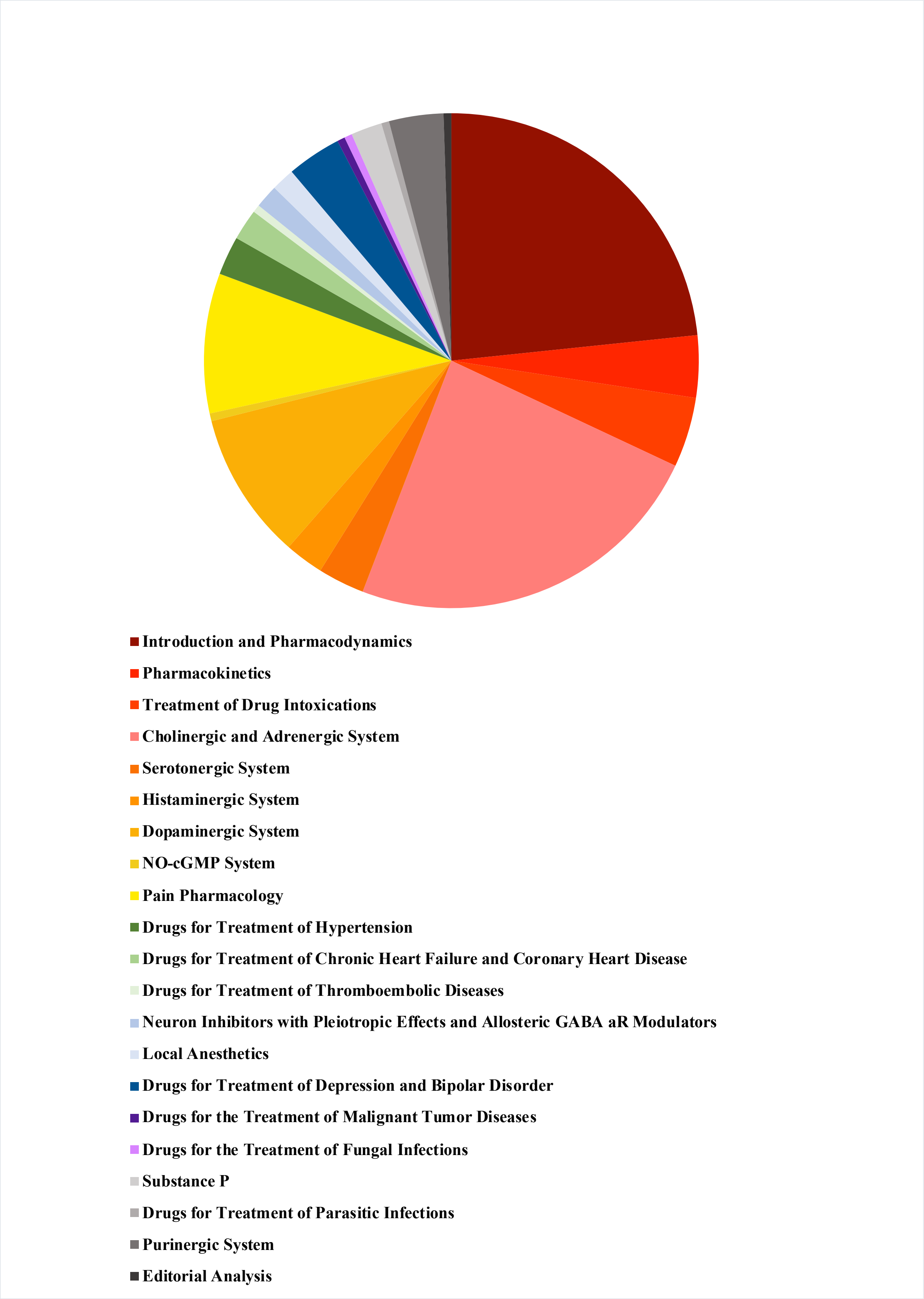

Fig. S8 Meta-data: 1980 Topics by chapters of "Basic Knowledge of Pharmacology"

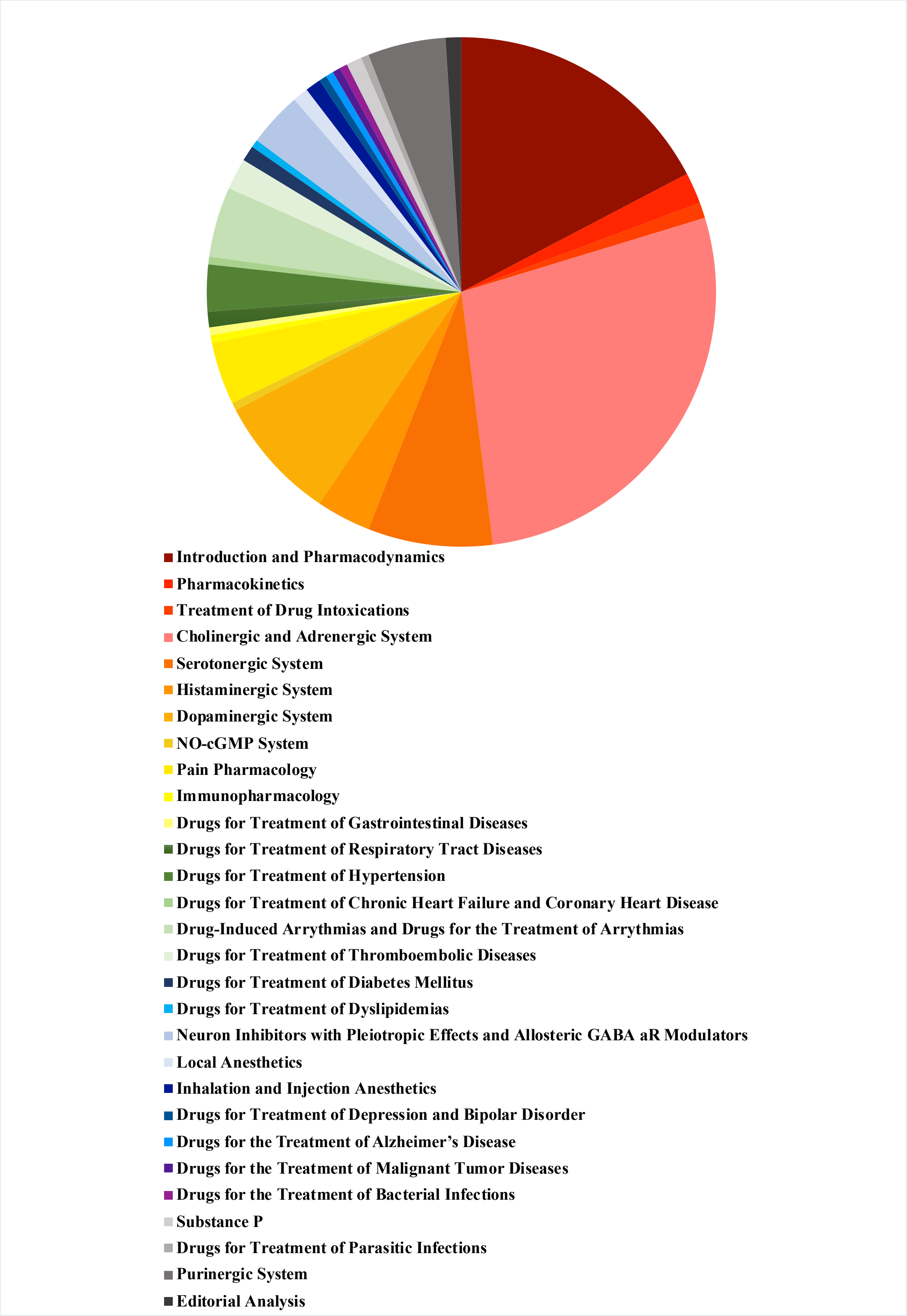

Fig. S9 Meta-data: 1990 Topics by chapters of "Basic Knowledge of Pharmacology"

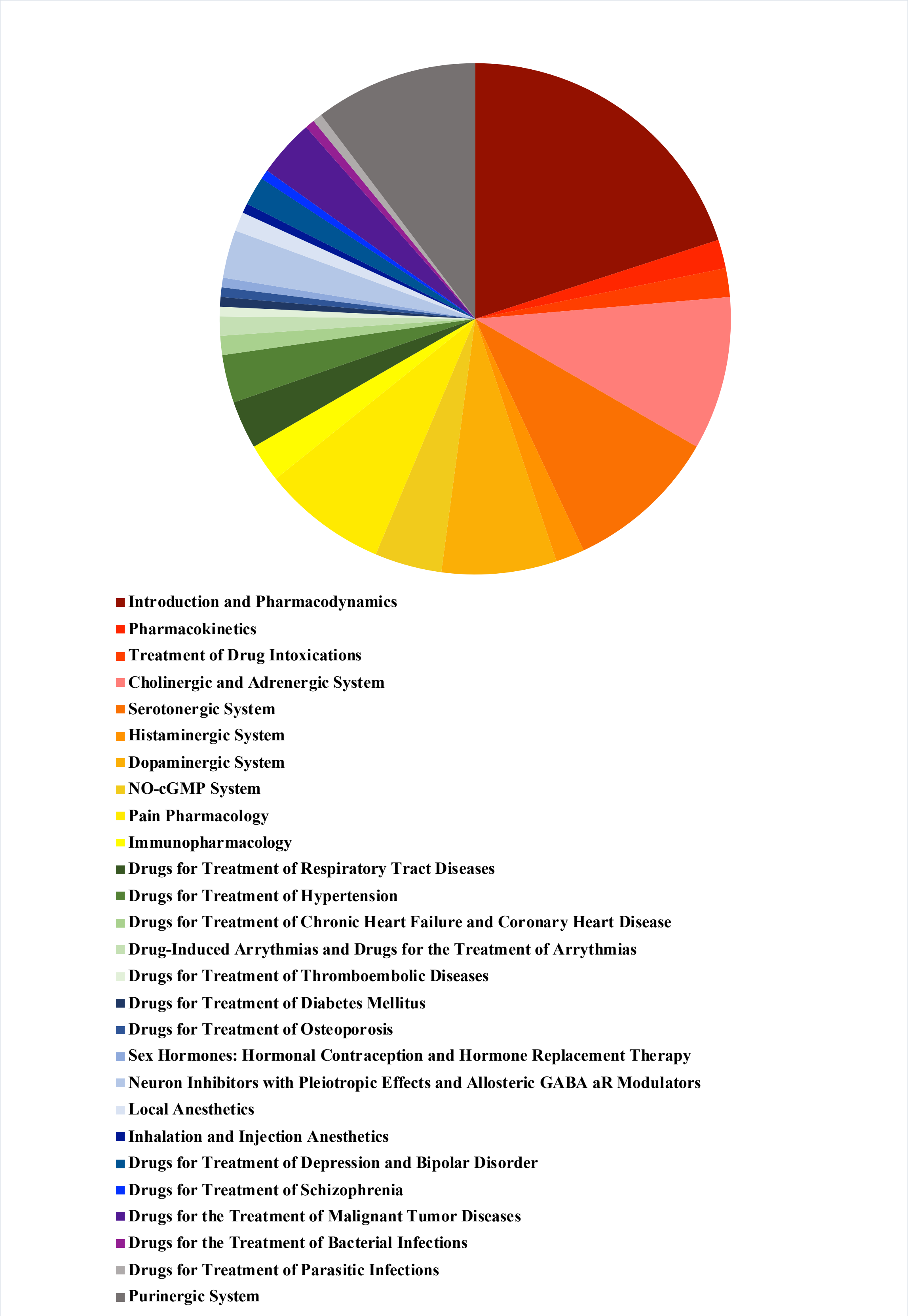

Fig. S10 Meta-data: 2000 Topics by chapters of "Basic Knowledge of Pharmacology"

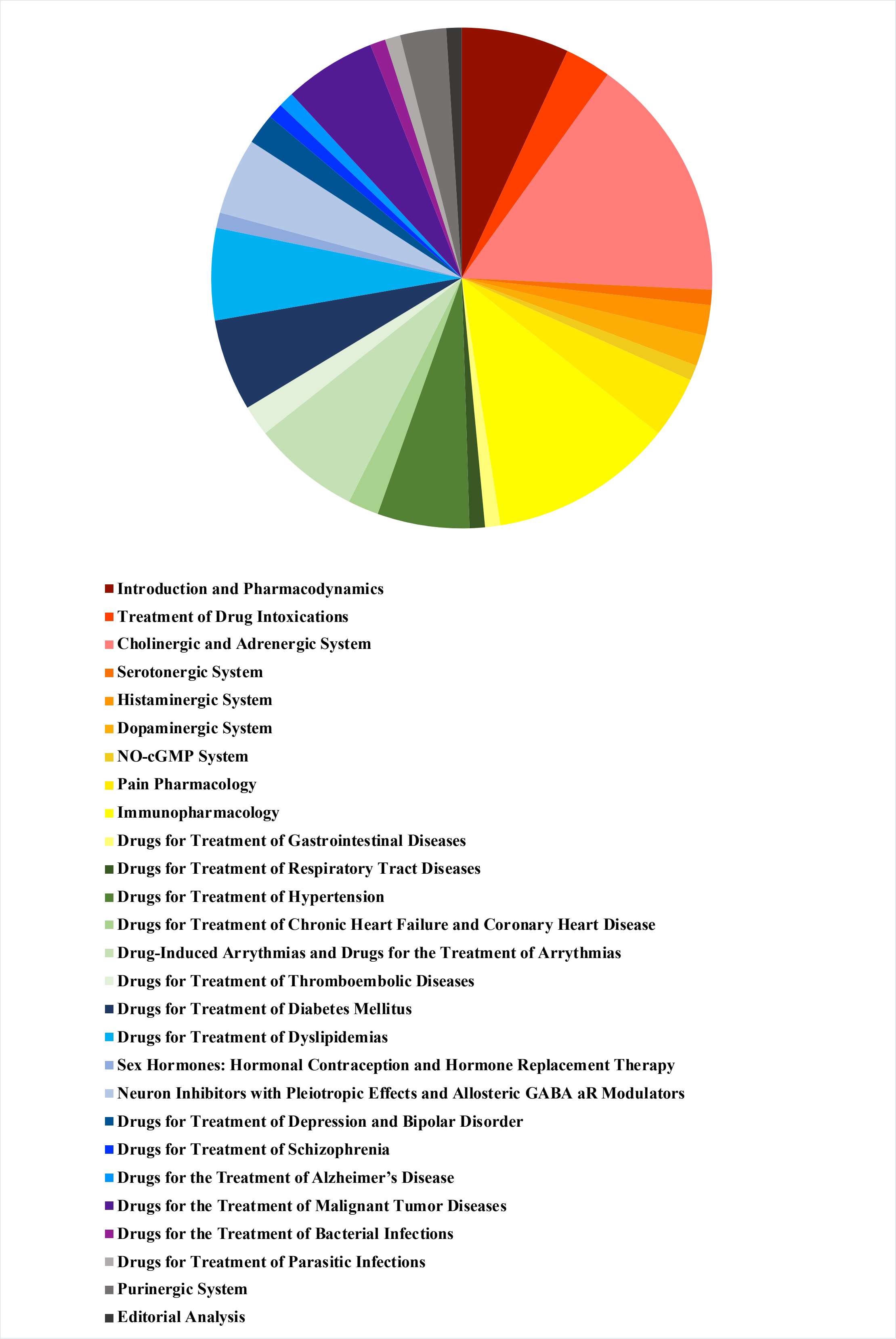

Fig. S11 Meta-data: 2010 Topics by chapters of "Basic Knowledge of Pharmacology"

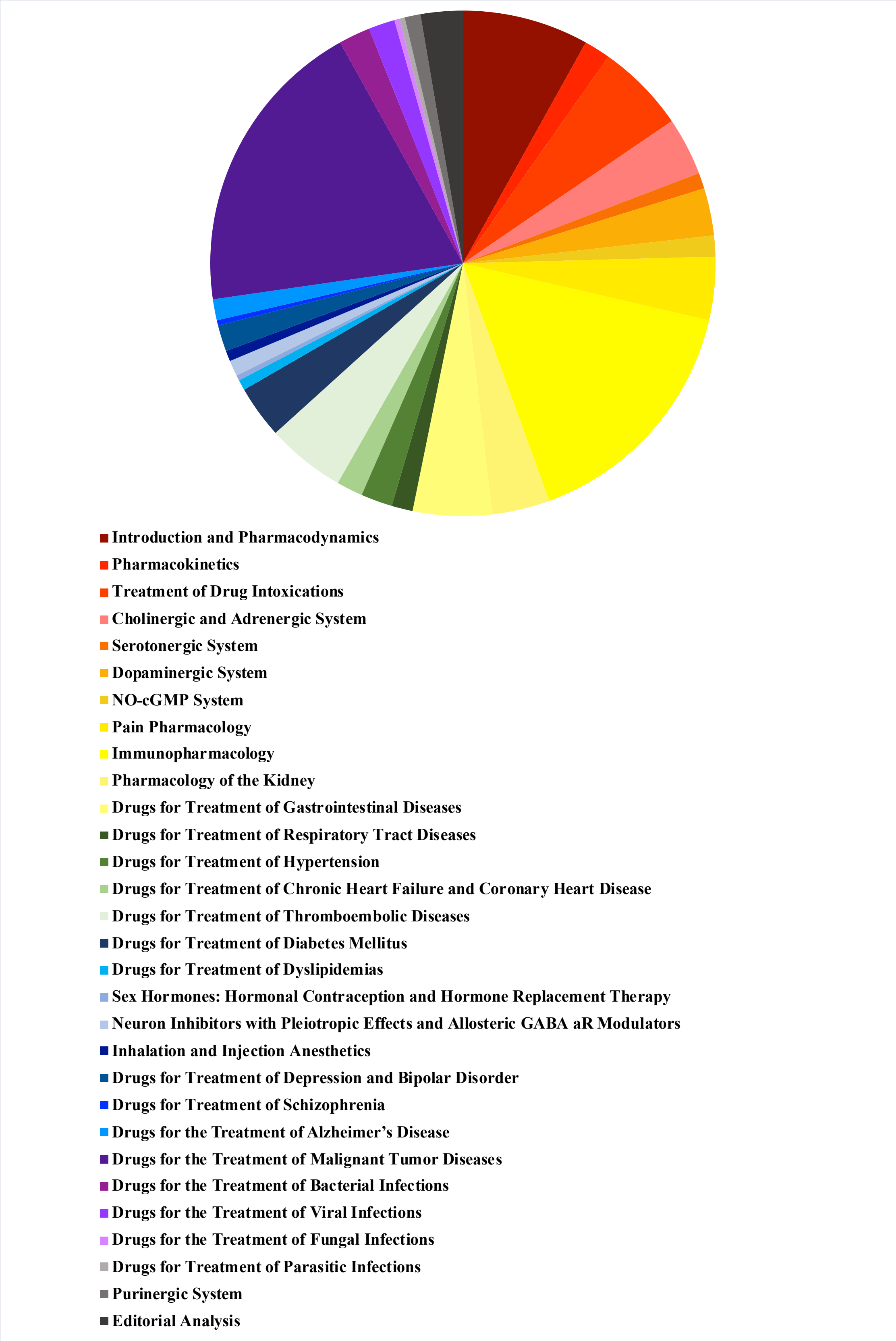

Fig. S12 Meta-data: 2020 Topics by chapters of “Basic Knowledge of Pharmacology

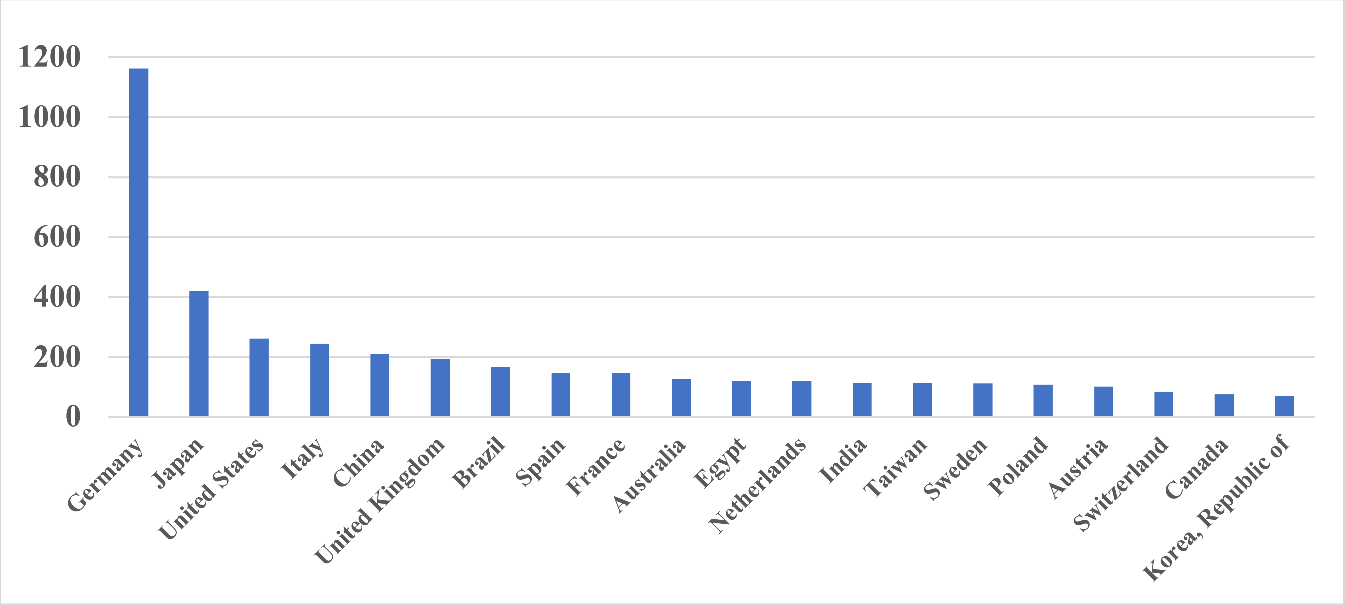

Fig. S13 Meta-data: Publications by the top-20 contributing countries (1990-2020)

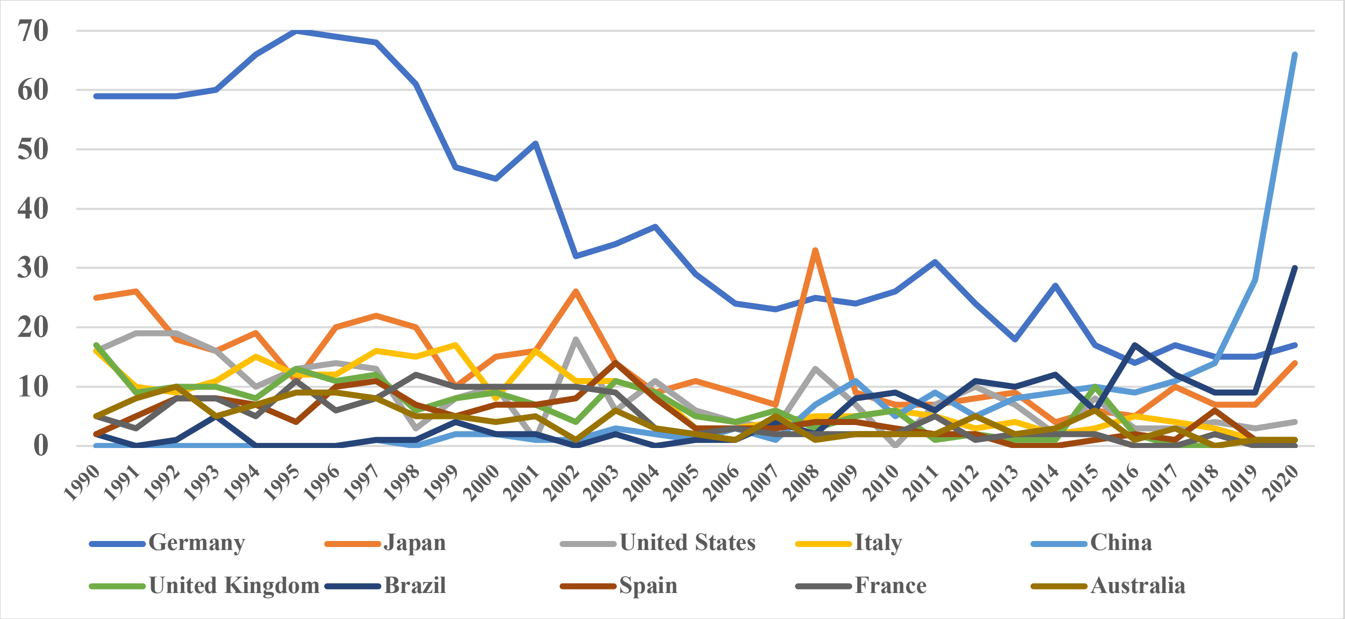

Fig. S14 Meta-Data: Publications by the TOP-10 contributing countries (1990-2020)

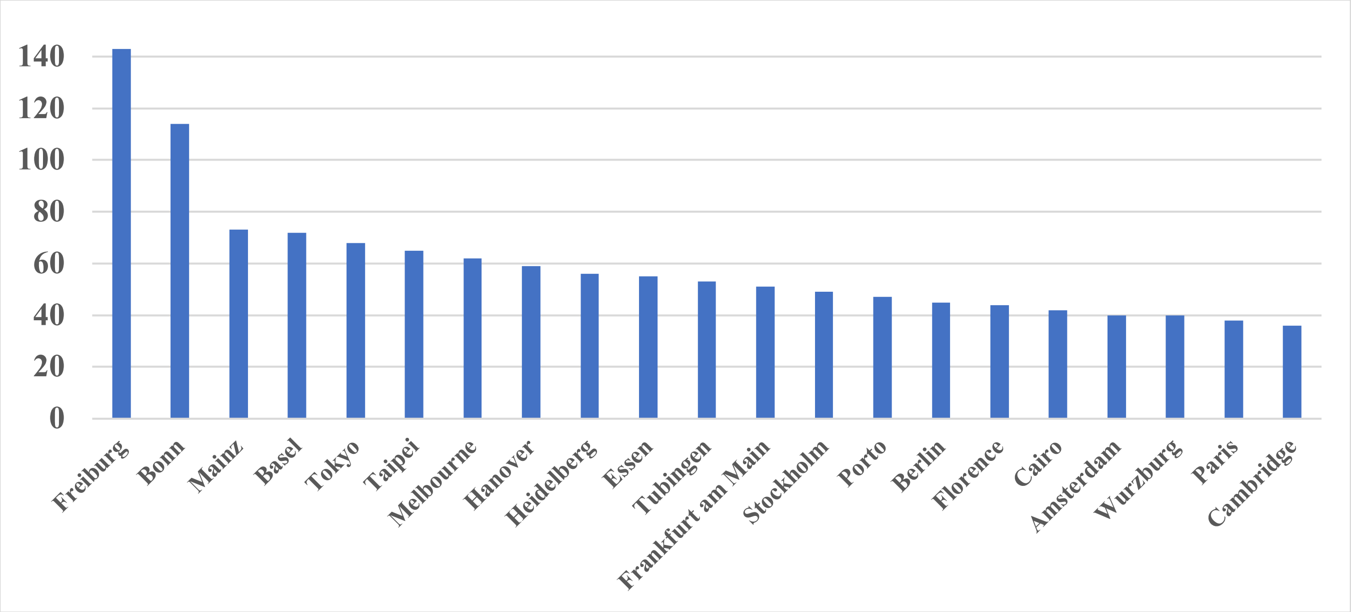

Fig. S15 Meta-data: Publications by the Top-20 contributing cities (1990-2020)
